# Proteotoxic stress disrupts epithelial integrity by inducing MTOR sequestration and autophagy overactivation

**DOI:** 10.1080/15548627.2022.2071381

**Published:** 2022-05-06

**Authors:** Xiaoxiang Cheng, Pei Zhang, Hongyu Zhao, Hui Zheng, Kai Zheng, Hong Zhang, Hongjie Zhang

**Affiliations:** a Centre of Reproduction, Development and Aging, Faculty of Health Sciences, University of Macau, Taipa, Macau, China; b National Laboratory of Biomacromolecules, Institute of Biophysics, Chinese Academy of Sciences, Beijing, China; c MoE Frontiers Science Center for Precision Oncology, University of Macau, Taipa, Macau SAR, China

**Keywords:** Autophagy, *C. elegans*, endosomal degradation, epithelial morphogenesis, LET-363/MTOR, proteostasis

## Abstract

Macroautophagy/autophagy, an evolutionarily conserved degradation system, serves to clear intracellular components through the lysosomal pathway. Mounting evidence has revealed cytoprotective roles of autophagy; however, the intracellular causes of overactivated autophagy, which has cytotoxic effects, remain elusive. Here we show that sustained proteotoxic stress induced by loss of the RING and Kelch repeat-containing protein C53A5.6/RIKE-1 induces sequestration of LET-363/MTOR complex and overactivation of autophagy, and consequently impairs epithelial integrity in *C. elegans*. In C53A5.6/RIKE-1-deficient animals, blocking autophagosome formation effectively prevents excessive endosomal degradation, mitigates mislocalization of intestinal membrane components and restores intestinal lumen morphology. However, autophagy inhibition does not affect LET-363/MTOR aggregation in animals with compromised C53A5.6/RIKE-1 function. Improving proteostasis capacity by reducing DAF-2 insulin/IGF1 signaling markedly relieves the aggregation of LET-363/MTOR and alleviates autophagy overactivation, which in turn reverses derailed endosomal trafficking and rescues epithelial morphogenesis defects in C53A5.6/RIKE-1-deficient animals. Hence, our studies reveal that C53A5.6/RIKE-1-mediated proteostasis is critical for maintaining the basal level of autophagy and epithelial integrity.

**Abbreviations:** ACT-5: actin 5; ACTB: actin beta; ALs: autolysosomes; APs: autophagosomes; AJM-1: apical junction molecule; ATG: autophagy related; C. elegans: Caenorhabditis elegans; CPL-1: cathepsin L family; DAF: abnormal dauer formation; DLG-1: Drosophila discs large homolog; ERM-1: ezrin/radixin/moesin; EPG: ectopic P granule; GFP: freen fluorescent protein; HLH-30: helix loop helix; HSP: heat shock protein; LAAT-1: lysosome associated amino acid transporter; LET: lethal; LGG-1: LC3, GABARAP and GATE-16 family; LMP-1: LAMP (lysosome-associated membrane protein) homolog; MTOR: mechanistic target of rapamycin kinase; NUC-1: abnormal nuclease; PEPT-1/OPT-2: Peptide transporter family; PGP-1: P-glycoprotein related; RAB: RAB family; RIKE-1: RING and Kelch repeat-containing protein; SLCF-1: solute carrier family; SQST-1: sequestosome related; SPTL-1: serine palmitoyl transferase family.

## Introduction

Polarized membrane transport, a process that sorts and delivers transmembrane proteins and lipids to either apical or basolateral membrane domains, is fundamental for the maintenance of epithelial polarity and integrity. As a sorting platform in this process, the endosomal system acts in both exocytic trafficking, by directing polarized proteins emanating from trans-Golgi network (TGN) to their destined membrane domains, and in endocytic trafficking, by recycling endocytosed cargos from the plasma membrane for redistribution or degradation [1–3]. Depending on the sorting signals they carry, some cargo proteins may require specific adaptors for their trafficking. As an example, some glycoproteins are recognized, sorted, and retained at the plasma membrane by galectins through the formation of galectin-glycan lattices [4,5].

The actin cytoskeleton plays an important role in polarized membrane transport. Canonically, actin filaments can function as tracks for the myosin-driven movement of vesicles [6,7], facilitating directional sorting. Alternatively, actin can also regulate membrane budding and fission through dynamin and/or CDC-42 and its downstream effectors, contributing to the biogenesis of transport carriers [8]. Finally, actin can also function to generate a local mechanical force, facilitating the movement of transport carriers [9].

Autophagy is a unique membrane trafficking process which is believed to contribute to epithelial homeostasis by converging with the endosomal system in multiple processes, including formation and expansion of the phagophore, biogenesis of autophagosomes (APs) and subsequent fusion of the APs with lysosomes (autolysosomes, ALs) for degradation [10–12]. Conceivably, endosome-mediated membrane trafficking will be affected by autophagy-dependent lysosomal degradation, and *vice versa*. Autophagy occurs at a basal level in most cells to maintain cellular homeostasis and fitness [13–16]. Moreover, autophagy can be induced by many types of stress, at least in part, through the inhibition of MTOR (mechanistic target of rapamycin kinase) [17]. The autophagic response needs to be tightly regulated because its level is often linked with beneficial or detrimental outcomes [18]. A persistent and overactivated autophagic response promotes organelle damage and cell death [19–22]. These effects can be partially restored by autophagy inhibition [18,21,23–25]. The stimuli that trigger autophagy overactivation and its cytotoxic effect *in vivo* remain largely unknown [26]. A mechanistic understanding of autophagy overactivation and its impact is critical to precisely control the level of autophagy for therapeutic intervention in many diseases [27–29].

Our exploration of the mechanisms underlying autophagy overactivation began with our interest in *C. elegans* intestinal development. Given that the actin cytoskeleton has diverse functions in polarized membrane trafficking and that multiple Kelch repeat family members have been shown to regulate cytoskeletal organization [30], we specifically investigated whether Kelch repeat-containing proteins modulate intestinal morphogenesis. Here we show that animals lacking the Kelch repeat-containing protein RIKE-1, encoded by a previously uncharacterized gene, *C53A5.6*, exhibit severe defects in intestinal morphology and integrity accompanied with mislocalization of intestinal membrane components and excessive endosomal degradation. We identify that autophagy overactivation is the underlying cause of excessive endosomal degradation. Blocking autophagosome formation prevents endosomal degradation and partially rescues intestinal morphology defects in animals with compromised RIKE-1 function. Further study reveals that autophagy overactivation is caused by imbalanced proteostasis, which consequently leads to cytoplasmic aggregation of proteins, including LET-363/MTOR. Attenuating the level of DAF-2 insulin/IGF1 signaling alleviates the LET-363/MTOR aggregation and autophagy overactivation, which in turn restores epithelial morphology in RIKE-1-deficient animals. Together, our findings suggest a role of RIKE-1 in maintaining the basal level of autophagy and epithelial integrity through shaping the cellular proteome.

## Results

### Loss of RIKE-1 results in defects in intestinal morphogenesis and integrity

**Figure 1.**
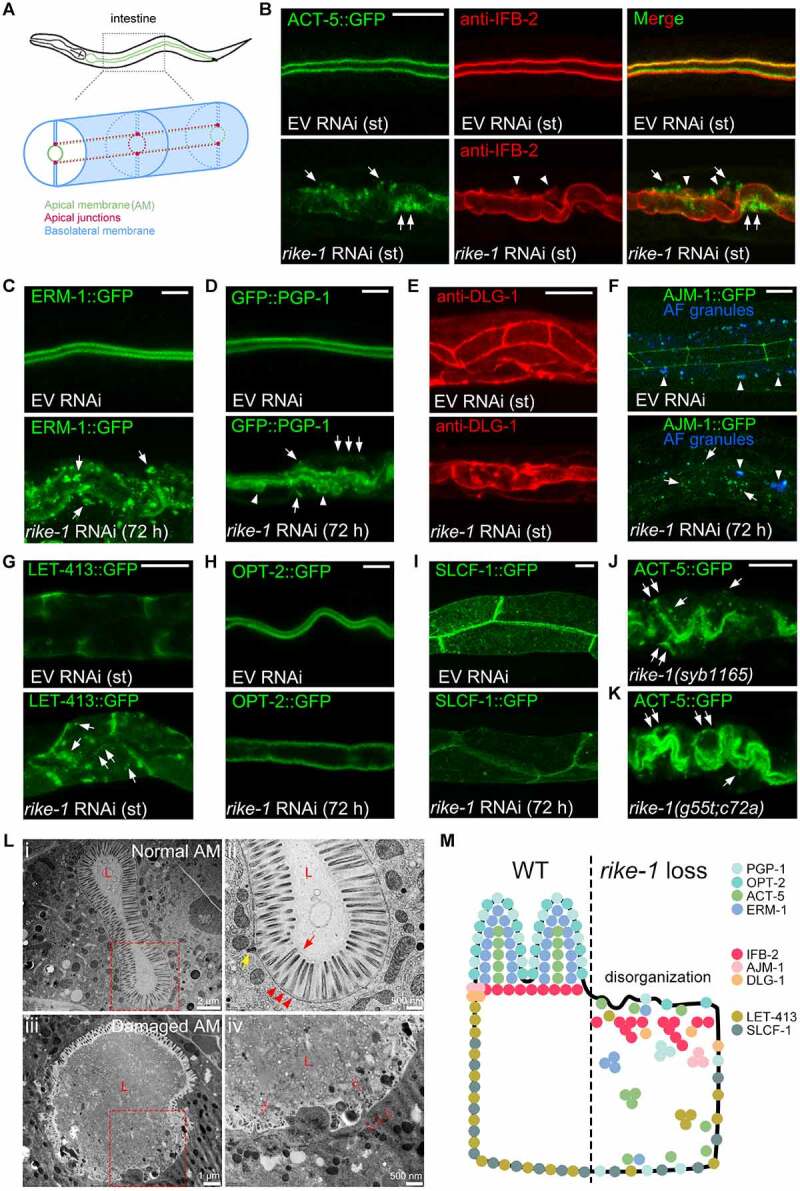
Loss of RIKE-1 results in defects in intestinal morphogenesis and integrity. (A) Schematic representation of polarized *C. elegans* intestinal cells. The apical membrane (green), apical junctions (red), and basolateral membrane (blue) are indicated. (B-J) Apical, junctional and basolateral membrane components in control (empty vector (EV) RNAi, upper panels) and RIKE-1-deficient (*rike-1* RNAi, lower panels) intestines. Here and below, st indicates that standard *rike-1* RNAi from parental L4 worms was performed and the arrested L1-stage F1 progeny were evaluated. For the rest, conditional (mild) RNAi initiated in L1-stage animals was performed and the arrested L3-L4 stage animals of the same generation were evaluated. Phenotypes were examined at 48 h or 72 h after the onset of RNAi, as indicated in the images. The same stages of control animals treated with EV RNAi were picked for imaging. (B) Apical actin labeled with ACT-5::GFP (green) and subapical intermediate filaments detected by an anti-IFB-2 antibody (red) exhibit apical detachment (arrows) and cytoplasmic aggregation (arrowheads). (C) Apical ERM-1, a lumenal scaffold component, is displaced and forms aggregates in cytoplasm (arrows). (D) Apical transmembrane P-glycoprotein PGP-1 is mispositioned basolaterally (arrows) accompanied with formation of ectopic bubble-like structures around the lumen (arrowheads). (E) The junctional belt distribution of DLG-1 is disorganized. (F) The junction integrity protein AJM-1 is totally displaced from the apicolateral junction and accumulates in cytoplasm (arrows). Arrowhead, auto-fluorescence granules. (G) Basolateral LET-413, which maintains apical distribution of junctional proteins, is mislocalized to the cytoplasm and apical domain (arrows). (H) OPT-2, an apical transmembrane oligopeptide transporter, remains apical. (I) The basolateral pyruvate transporter SLCF-1 remains basolateral. (J and K) *rike-1* deletion- (J) and pre-stop- (K) mutants phenocopy *rike-1* RNAi animals, as characterized by mislocalization of ACT-5::GFP (arrows). Scale bars: 10 μm for all confocal images. (L) TEM micrographs of intestinal cross-sections showing normal (i,ii) and disrupted structures (iii,iv) in *rike-1* RNAi larvae. High-magnification view images of intestinal structures (red dotted boxes in i,iii) are shown in ii,iv. The oval lumen (l) with dense microvilli (red arrows), terminal web (red arrowheads), and intact apical junctions (yellow arrows) are observed in normal intestine (ii). *rike-1* RNAi causes damaged and shortened microvilli (red dotted arrows), and disappearance of the terminal web (red dotted arrowheads) and apical junctions (iv). (M) Schematic summarizing defective distribution of intestinal membrane components in RIKE-1-deficient intestinal cells.

To investigate possible roles of Kelch repeat-containing proteins in modulating intestinal morphogenesis, we carried out RNAi knockdown of 11 *C. elegans* proteins containing Kelch repeats (based on the SMART database) using worms expressing the lumenal membrane marker ACT-5::GFP. We identified that the previously uncharacterized gene *C53A5.6* shows fully penetrant L1 larval lethality with severe intestinal morphogenesis defects upon standard RNAi treatment [31] (Figure 1 and Table S1). The protein encoded by *C53A5.6* is distinct from other Kelch repeat proteins owing to the presence of an N-terminal RING domain; therefore, we named this gene *rike-1* (RING- and Kelch-containing protein). Given the severe phenotypes caused by standard *rike-1* RNAi knockdown where RNAi is initiated in L4-stage worms and the progeny are evaluated for phenotypes, most of the RNAi experiments were performed using a conditional knockdown approach where RNAi is initiated from L1s and the same generation animals are scored. Detailed analysis revealed that loss of RIKE-1 upon standard or mild RNAi caused cytoplasmic mislocalization and aggregation of multiple apical membrane-associated proteins, including the cortical actin ACT-5, the membrane-cytoskeleton linker ERM-1, the intermediate filament protein IFB-2, and the integral membrane protein PGP-1 (Figure 1B-D). The apical junction component DLG-1 was disorganized (Figure 1E), whereas AJM-1 entirely disappeared from the apicolateral junctures and accumulated in the cytoplasm (Figure 1F) in intestinal cells of *rike-1(RNAi)* animals. Moreover, the basolateral protein LET-413/SCRIB formed cytoplasmic aggregates with partial displacement to the subapical domain (Figure 1G). Of note, the distribution of the apical peptide transporter PEPT-1/OPT-2 and the basolateral pyruvate transporter SLCF-1 was less affected (Figure 1H,I). Transmission electron microscopy (TEM) analysis revealed striking lumenal structural defects. These included loss of microvilli and disruption of the terminal web, a cytoskeletal structure on the apical surface of intestinal cells (Figure 1L and S1A). These results suggest that loss of *rike-1* causes intestinal atrophy and defects in assembly and maintenance of intestinal membrane components at designated positions (Figure 1M).

To confirm the role of RIKE-1 in intestinal development, we used CRISPR-mediated genome editing to generate two germline mutants: *rike-1(syb1165)*, which carries a 2140 bp-deletion of the *rike-1* sequence, and *rike-1(g55t;c72a)*, which carries two premature stop codons (Figure S1B and S1C). Consistent with the RNAi results, the two RIKE-1 loss-of-function mutants showed early larval lethality accompanied with mislocalization of ACT-5::GFP (Figure 1J,K). Furthermore, intestine-, but not muscle- or epidermis-specific RNAi caused larval lethality (Figure S1D). These results further demonstrated that RIKE-1 is required for larval development and intestinal morphogenesis.

### Loss of RIKE-1 promotes endosomal enlargement and fusion with lysosomes

**Figure 2.**
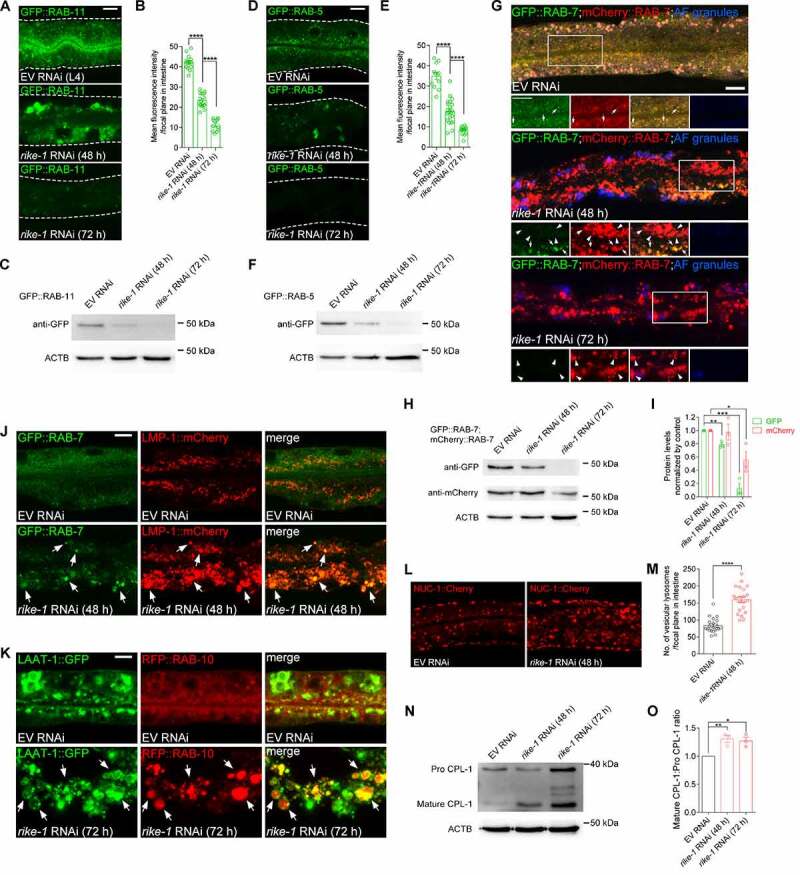
Loss of RIKE-1 promotes endosomal enlargement and fusion with lysosomes. (A-I) Morphometric and quantitative analysis of endosomal markers in control, *rike-1* RNAi (48 h) and *rike-1* RNAi (72 h) larvae. (A-C) *rike-1* RNAi causes enlargement of recycling endosomes labeled with recycling endosome marker GFP::RAB-11 at 48 h; this is followed by a significant decline of GFP fluorescence at 72 h (A). Quantification of fluorescence intensity is shown in (B) (*n* = 14,18,12). Western blot of GFP::RAB-11 is shown in (C). (D-F) *rike-1* RNAi results in enlargement of early endosomes labeled with early endosome marker GFP::RAB-5 at 48 h; this is followed by a significant decline of GFP fluorescence at 72 h (D). Quantification of fluorescence intensity is shown in (E) (*n* = 12,21,15). Western blot of GFP::RAB-5 is shown in (F). (G-I) In contrast to small and evenly distributed late endosomal GFP::RAB-7 and mCherry::RAB-7 (arrows; yellow puncta) in control animals, *rike-1* RNAi induces enlargement of both GFP- and Cherry-tagged RAB-7 endosomes, accompanied with a dramatic decrease of GFP signals (G). Arrows, RAB-7 endosomes emitting both GFP and Cherry fluorescence; arrowheads, RAB-7 endosomes emitting Cherry signal only. AF: autofluorescence. Western blots of GFP::RAB-7 and mCherry::RAB-7 are shown in (H), with quantification in (I). (J and K) Colocalization of endosomes with lysosomes. In *rike-1* RNAi worms, enlarged late endosomes GFP::RAB-7 are colocalized with LMP-1::mCherry-labeled lysosomes (J, arrows) and enlarged recycling endosomes (labeled by RFP::RAB-10) are enclosed in LAAT-1::GFP-labeled lysosomes (K, arrows). Three biologically independent RNAi experiments were performed with similar results (*n* > 20 animals). (L and M) *rike-1* RNAi leads to increased number of vesicular lysosomes labeled by NUC-1::cherry in intestine, with quantification in (M) (*n* = 21 each). (N and O) Western blot analysis of CPL-1 processing in control and *rike-1* RNAi group (N). The ratio of the mature CPL-1 versus pro CPL-1 was quantified (O). All statistical analyses were performed using two-tailed unpaired *t*-tests. Error bars indicate mean ± SEM. * *P* < 0.05, **P < 0.01, ***P < 0.001, ****P < 0.0001, ns, not significant. Scale bars: 10 μm.

In intestinal epithelial cells, apicobasal polarity and integrity are maintained through constant, polarized trafficking of both protein and lipids [32,33]. Loss of microvilli and mislocalization of membrane components is often associated with disordered endosomal trafficking [34,35]. Indeed, we observed that loss of the apical recycling endosomal component RAB-11 induced the mislocalization of ACT-5::GFP (Figure S2A). We therefore examined the effect of *rike-1* knockdown on endosomal markers. Intriguingly, all types of endosomes examined, including RAB-5-labeled early endosomes (EEs), RAB-7-marked late endosomes (LEs), and RAB-10-, RME-1- and RAB-11-positive recycling endosomes (REs), were dramatically enlarged and clustered in intestinal cytoplasm at an early stage (48 h; see Materials and Methods for staging) after *rike-1* RNAi exposure (Figure 2A-I and S2B-D). Unexpectedly, we found a strong reduction in fluorescence of GFP-labeled endosomes at a later stage (72 h) after *rike-1* RNAi (Figure 2A-G). Compared with mCherry signals, GFP fluorescence is easily quenched in the acidic lysosomal environment [36,37], so this result suggests that the abnormally enlarged endosomes were likely fused with lysosomes. As expected, we observed strong colocalization of GFP-positive endosomal clusters with the lysosomal marker LMP-1::mCherry in early stage (48 h) *rike-1(RNAi)* animals (Figure 2J and S2E). Moreover, the enlarged RAB-7 LEs and RAB-10 REs were enclosed within LAAT-1::GFP-labeled lysosomes in *rike-1*-deficient worms at the later stage (72 h) (Figure 2K and S2F). Of note, LAAT-1::GFP was not easily quenched like the endosomal GFP signals, because LAAT-1 is localized to lysosomal membranes [38]. These data indicate that endosome-lysosome fusion upon RIKE-1 loss facilitates endosomal degradation. Several lines of evidence further support this notion. First, we detected a significant decrease of protein levels of RAB-5, RAB-7 and RAB-11 in late-stage *rike-1(RNAi)* animals (Figure 2A-I). Second, we observed dramatic degradation of the GFP-tagged recycling cargo IL2RA/hTAC in *rike-1*-deficient worms (Figure S2G and 2H). Third, in *rike-1(RNAi)* animals, both the number of vesicular lysosomes and the total volume of lysosomes tagged by NUC-1::Cherry increased significantly (Figure 2L,M). Finally, we examined the processing of CPL-1 (CathePsin L family) from the inactive propeptide to the active mature form, which is an indicator for the degradation activity of lysosomes [39]. We found that more mature CPL-1 was produced in *rike-1*-deficient worms (Figure 2N,O). Together, these results suggest that RIKE-1 loss promotes enlargement of trafficking endosomes and likely forces them to undergo lysosomal degradation.

### Loss of RIKE-1 induces autophagy activation

**Figure 3.**
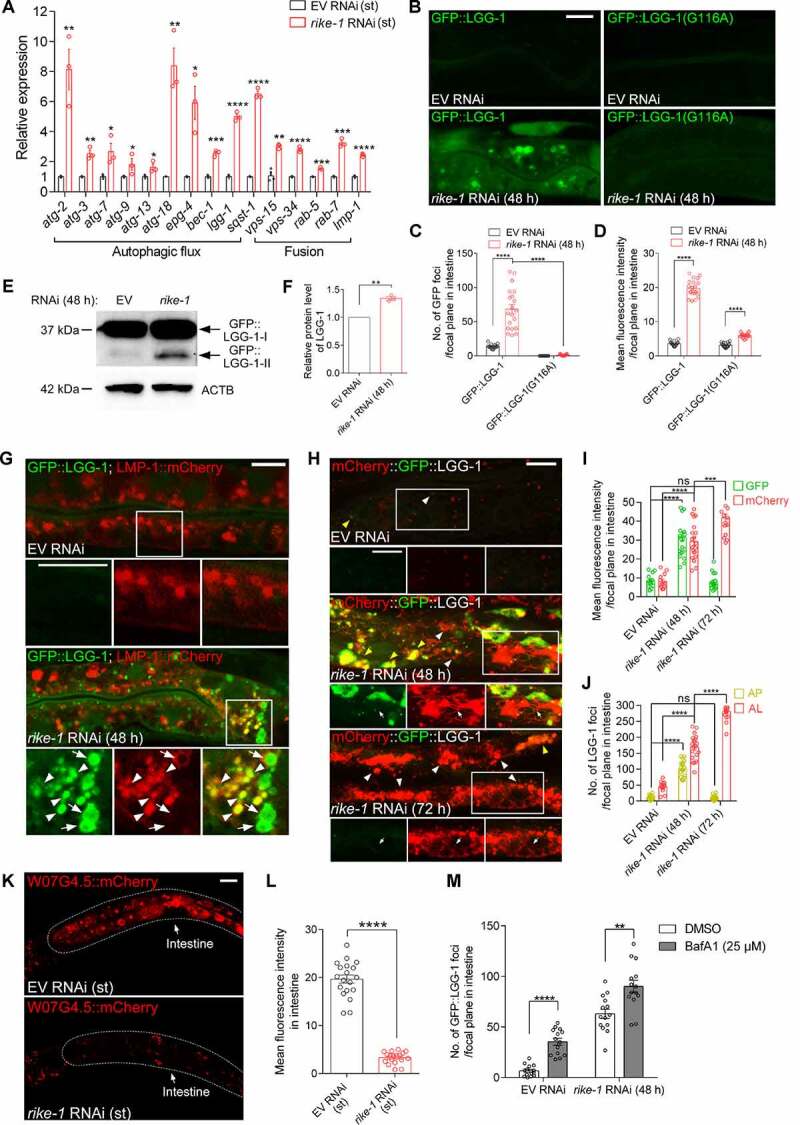
Loss of RIKE-1 induces autophagy activation. (A) Relative expression of autophagy-related genes measured by qRT–PCR in *rike-1* RNAi-treated L1 larvae versus control L1 larvae. The mRNA levels were normalized to *tba-1. n* = 3 independent biological replicates. (B-D) *rike-1* RNAi causes increased number and intensity of GFP-positive autophagosomes in animals expressing GFP::LGG-1, but not in animals expressing GFP::LGG-1(G116A). Quantification of the number of GFP puncta (arrows) and fluorescence intensity is shown in (C) and (D) (*n* = 18 each). (E and F) Western blot of LGG-1-I and PE-conjugated LGG-1-II in control and *rike-1* RNAi (48 h) larvae (E), with quantification of the total LGG-1 protein level (F). (G) Colocalization of autophagosomes labeled by LGG-1::GFP with lysosomes marked by LMP-1::mCherry in control and *rike-1* RNAi larvae. Arrows, autophagosomes; arrowheads, autolysosomes. (H-J) Intestinal expression of the dual-fluorescent mCherry::GFP::LGG-1 reporter in control, *rike-1* RNAi (48 h) and *rike-1* RNAi (72 h) larvae (H). Quantification of fluorescence intensity is shown in (I) (*n* = 13,20,17; ns, *P* = 0.6728). Quantification of the number of autophagosomes (APs, yellow arrowheads) and autolysosomes (ALs, white arrowheads) in (J) (*n* = 13,20,17; ns, *P* = 0.5834). (K and L) The intestinal fluorescence intensity of W07G4.5::mCherry is reduced in *rike-1* RNAi larvae (K); quantification is shown in (L) (*n* = 19,18). (M) *rike-1* RNAi further increases the number of intestinal GFP::LGG-1 puncta in the presence of Bafilomycin A1. For all panels, three biologically independent RNAi experiments were performed with similar results. All statistical analyses were performed using two-tailed unpaired *t*-tests. Error bars indicate mean ± SEM. **P < 0.01, ***P < 0.001, ****P < 0.0001, ns, not significant. Scale bars: 10 μm.

The seemingly excessive endosomal-lysosomal degradation in RIKE-1-deficient animals prompted us to examine the expression of genes implicated in autophagy and other related pathways. By using the quantitative reverse transcription polymerase chain reaction (qRT-PCR), we found significant upregulation of the genes involved in autophagosome biogenesis (*atg-2, atg-18, epg-4* and *lgg-1*) and fusion with lysosomes (*vps-34, rab-7* and *lmp-1*) in *rike-1* RNAi animals and/or germline mutants (Figure 3A and S3A). We used a transgenic strain expressing GFP-tagged LGG-1, the *C. elegans* ortholog of mammalian Atg8/LC3, to label autophagosomes (APs). In *rike-1*-deficient worms, GFP::LGG-1 was highly expressed and formed abnormally enlarged puncta (Figure 3B-D and S3B-D). During autophagy, the cytosolic protein LC3 (LC3-I) is conjugated to phosphatidylethanolamine (PE) to generate LC3-II, which is subsequently incorporated into phagophores. After fusion with endosomes, mature APs are eventually delivered to lysosomes to form autolysosomes (ALs) for degradation of substrates. To further confirm that the enlarged GFP::LGG-1 puncta were lipidated autophagosomes rather than aggregates, we examined worms expressing the lipidation-deficient GFP::LGG-1(G116A) mutant. Notably, GFP::LGG-1(G116A) failed to form puncta in the intestine of *rike-1(RNAi)* animals (Figure 3B-D). Moreover, *rike-1* RNAi animals showed a marked increase in the endogenous LC3-II level (Figure 3E,F). Together, these results indicate that autophagy is hyperactivated in *rike-1*-deficient worms.

Elevated autophagy activity could result from accelerated autophagosome biogenesis and/or blockade of the downstream autophagosome-lysosome fusion and degradation events. To distinguish between the two possibilities, we examined the fusion of autophagosomes with late endosomes and lysosomes. Strong colocalization of RAB-7, RAB-10 and LMP-1 with enlarged GFP::LGG-1 puncta was observed (Figure 3G, S3E and S3F), suggesting that autophagosome-lysosomes fusion is not compromised. To further corroborate these findings, we used a tandem-tagged mCherry::GFP::LGG-1 reporter that labels APs yellow (positive for both GFP and mCherry) and acidic ALs red (positive for mCherry only, due to the GFP signal being quenched in an acidic environment) [37]. At the early stage of *rike-1* RNAi (48 h), the number and intensity of APs (yellow [green/red] puncta) and ALs (red puncta) were significantly elevated, and tubular structures extruding from ALs were also observed (Figure 3H-J). Later (72 h), only ALs (red puncta) were detected (Figure 3H-J), indicating a normal degradation process. Moreover, we tested the effect of RIKE-1 loss on degradation of autophagy substrates [40]. The intestinal level of W07 G4.5::mCherry was significantly reduced in *rike-1(RNAi)* animals (Figure 3K,L). Another target of autophagy, the SQSTM1/p62 homolog SQST-1, accumulates in *rpl-43* mutants owing to impaired protein synthesis and can be removed by autophagy activation [41]. The number of SQST-1::GFP puncta was also significantly reduced at 72 h after *rike-1* RNAi (Figure S3G and S3H). It is worth noting that there is no contradiction between the observed degradation of GFP::LGG-1 and SQST-1::GFP puncta at 72 h after conditional *rike-1* RNAi initiated from the L1 stage and the observed upregulation of LGG-1 and SQST-1 in arrested L1s under standard RNAi. Rather, these results respectively reflect a mild and prolonged response versus a strong and acute response. Last, to measure autophagic flux, we treated the worms with the lysosomal inhibitor bafilomycin A_1_, and found that bafilomycin A_1_ treatment led to a further increase in the number of GFP::LGG-1 puncta (Figure 3M). Together, these findings argue strongly for an overactivated autophagic flux under *rike-1* RNAi.

To rule out the possibility that autophagy overactivation is secondary to lethality, or defective epithelial morphogenesis and/or membrane trafficking, we knocked down several essential genes with known function in intestinal morphogenesis and/or trafficking, such as *act-5* [42], *sptl-1* [43] and *rab-11* [44], to check whether they cause enlarged APs. As expected, knocking down each of these genes resulted in highly penetrant larval arrest phenotypes, but not in the accumulation of enlarged GFP::LGG-1 puncta (Figure S3I). This provides further evidence that loss of RIKE-1 specifically induces autophagy upregulation.

### Autophagy overactivation induced by RIKE-1 loss causes cytotoxicity

**Figure 4.**
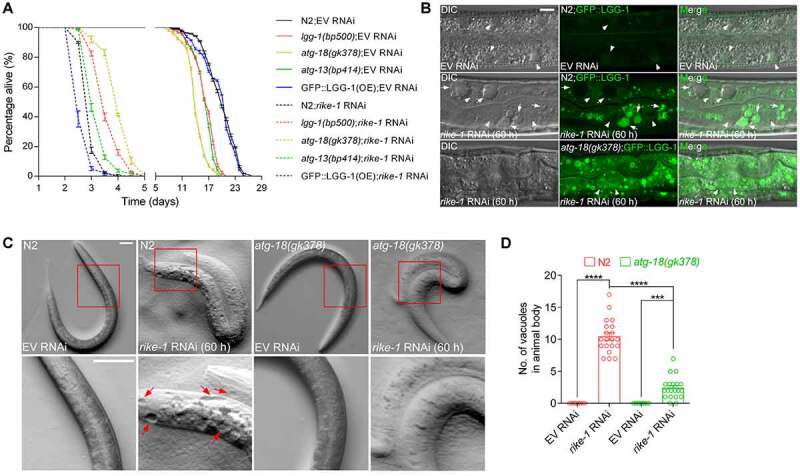
Autophagy overactivation induced by RIKE-1 loss causes cytotoxicity. (A) Mutations in the autophagy-related genes *lgg-1, atg-18* and *atg-13* partially rescue the short lifespan induced by *rike-1* RNAi while LGG-1::GFP overexpression further shortens the lifespan in *rike-1* RNAi animals. *n* = 5 independent biological replicates. Of note, *lgg-1(bp500)* and *atg-18(gk378)* mutants show larval lethality; escapers were used for lifespan analysis. (B) DIC and fluorescence images and their overlay in control and *rike-1* RNAi (60 h) larvae expressing GFP::LGG-1. *rike-1* RNAi generates transparent vacuoles (arrows) in intestine, which is suppressed by *atg-18(gk378)* mutation. Arrowheads, nuclei. Scale bar: 10 μm. (C and D) The big vacuoles along the animal body observed in *rike-1(RNAi)* larvae are suppressed by *atg-18(gk378)* mutation (C). Quantification of vacuole numbers is shown in (D). *n* = 10 for EV RNAi groups; *n* = 20 for *rike-1* RNAi groups. Scale bar: 50 μm. All statistical analyses were performed using two-tailed unpaired *t*-tests. Error bars indicate mean ± SEM. ***P < 0.001, ****P < 0.0001. ns, not significant.

We next investigated whether the hyperactivated autophagy induced by RIKE-1 loss causes cytotoxicity or boosts survival. Mild *rike-1* RNAi initiated from L1 stage resulted in L3-L4 stage larval lethality, with a maximum lifespan of 96 hours. Mutations of autophagy-related genes, including *lgg-1(bp500), atg-18(gk378)* and *atg-13(bp414)*, led to shortened lifespan in the control group of animals (Figure 4A), as previously reported [45]. However, they partially extended the lifespan of *rike-1*-deficient worms whereas GFP::LGG-1 overexpression further shortened the lifespan (Figure 4A). This implies that autophagy has a detrimental effect upon *rike-1* knockdown. ATG-18 encodes a *C. elegans* homolog of human WIPI (WD repeat domain, phosphoinositide interacting) proteins [46]. ATG-18 is thought to promote LC3 lipidation and AP formation through binding to phosphatidylinositol 3-phosphate/PtdIns3P and recruiting LC3/ATG12–ATG-5/ATG5-ATG-16.1/ATG16 L1 complex like its mammalian homolog [47]. In *atg-18(gk378)* mutants, the number and intensity of GFP::LGG-1 puncta were slightly higher than in wild-type animals, probably representing accumulation of phagophores. When *atg-18(gk378)* mutants were subjected to RNAi depletion of RIKE-1, the number and intensity of GFP::LGG-1 puncta were greatly increased, similar to the phenotype observed in *rike-1(RNAi)* animals; however, the puncta persisted without further degradation due to impaired autophagy activity (Figure S4A-C). Cytoplasmic vacuolization is one of the known characteristics of autophagy-dependent cell death. RIKE-1 loss generated many vacuoles in intestinal cells and along the body; this vacuolization was significantly suppressed in *atg-18(gk378)* mutants (Figure 4B-D). These results suggest that hyperactivated autophagy caused by RIKE-1 loss is cytotoxic.

### Inhibition of autophagy prevents endosomal degradation and partially restores intestinal morphology in RIKE-1-deficient animals

**Figure 5.**
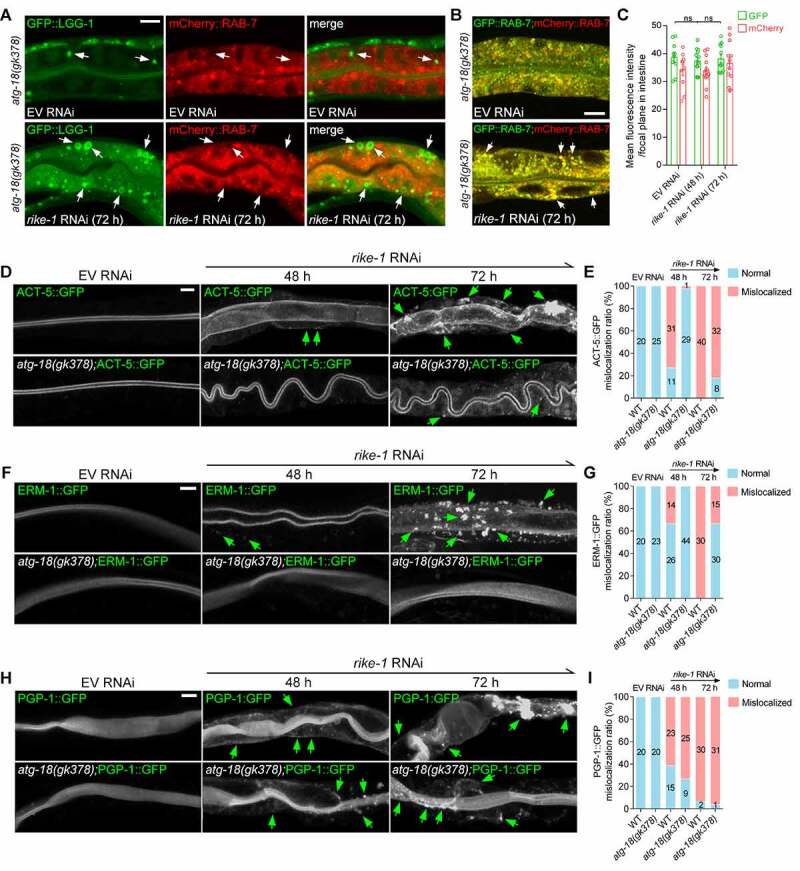
Inhibition of autophagy prevents endosomal degradation and partially restores intestinal morphology and growth in RIKE-1-deficient animals. (A) *atg-18(gk378)* mutation prevents excessive fusion of GFP::LGG-1-positive puncta (arrows) with mCherry::RAB-7 clusters (see Figure S3E for comparison). Three biologically independent RNAi experiments were performed with similar results (*n* > 20 animals). (B and C) *atg-18(gk378)* mutation suppresses GFP::RAB-7 degradation induced by *rike-1* RNAi (b); quantification of fluorescence intensity is shown in (C) (see Figure 2G for comparison, *n* = 10,13,12; ns, not significant; *P* = 0.4968, 0.7697). Error bars indicate mean ± SEM. (D-I) *atg-18(gk378)* mutation partially alleviates the mislocalization of ACT-5::GFP (D) and ERM-1::GFP (F), but not PGP-1::GFP (H), caused by *rike-1* RNAi. (E, G, and I) Quantification of mislocalization phenotypes of ACT-5::GFP, ERM-1::GFP and PGP-1::GFP in *rike-1(RNAi)* and *atg-18(gk378);rike-1(RNAi)* animals. *n* values are indicated in the figures from three biologically independent RNAi experiments. Scale bars: 10 μm.

To confirm that hyperactivated autophagy is the cause of the excessive endosomal-lysosomal degradation in *rike-1*-deficient worms, we tested whether autophagy inhibition can prevent endosomal degradation. We found that, unlike in *rike-1(RNAi)* animals, most of the LGG-1 vesicles were not colocalized with RAB-7 in *atg-18(gk378);rike-1(RNAi)* mutants (Figure 5A vs. Figure S3E). Moreover, the fluorescence intensity of GFP::RAB-7 was not diminished at all in *atg-18(gk378);rike-1(RNAi)* mutants (Figure 5B,C vs. Figure 2G). This indicates that the degradation of RAB-7-labeled LEs induced by RIKE-1 loss was completely suppressed upon autophagy inhibition. Similarly, the apical distribution of RAB-11-labeled endosomes was partially preserved in *atg-18(gk378)* mutants (Figure S4D). Suppression of the *rike-1* (RNAi)-induced endosomal degradation by *atg-18(gk378)* mutation could also be judged by the signal from GFP-tagged RAB-10 and RAB-11 (Figure S4E and S4F). These data suggest that in *rike-1*-deficient worms, hyperactivated autophagy hijacks endosomes to facilitate degradation of cytoplasmic content.

Considering that excessive degradation of endosomes may underlie the intestinal morphology defects in *rike-1*-deficient worms, we next examined whether the disrupted intestinal morphogenesis is also relieved upon autophagy inhibition. A striking restoration of lumen width was observed in *rike-1*-deficient worms carrying the *atg-18(gk378)* mutation (Figure S4G). The mislocalization of ACT-5::GFP in *rike-1(RNAi)* animals was partially alleviated by *atg-18(gk378)* mutation (Figure 5D,E). Similar rescuing effects were also observed for ERM-1 and AJM-1 (Figure 5F,G, S4H and S4I). However, the mislocalization of p-glycoprotein PGP-1 was not rescued in response to autophagy inhibition (Figure 5H,I). The discrepancy in the rescuing effects on the mislocalization of PGP-1 and other apical domain components (such as ACT-5, ERM-1 and AJM-1) lies in the fact that PGP-1 is an integral membrane protein, and its sorting and transport may also depend on the interaction of its transmembrane domains with sorting receptors and adaptors or direct partitioning into lipid domains [48] in addition to endosomes. Support for this explanation comes from a previous study showing that PAR-5 depletion alters the apical localization of F-actin and recycling endosome RAB-11, but has no effect on the localization of the apical membrane proteins PGP-1 and PEPT-1 [44]. This suggests that RIKE-1 probably acts to maintain PGP-1 at the apical membrane through an autophagy/endosome-independent mechanism. Thus, RIKE-1 loss leads to excessive degradation of endosomes due to hyperactivated autophagy, which, in turn, misroutes membrane components and disrupts epithelial integrity.

### RIKE-1 loss results in proteostasis imbalance and MTOR aggregation

**Figure 6.**
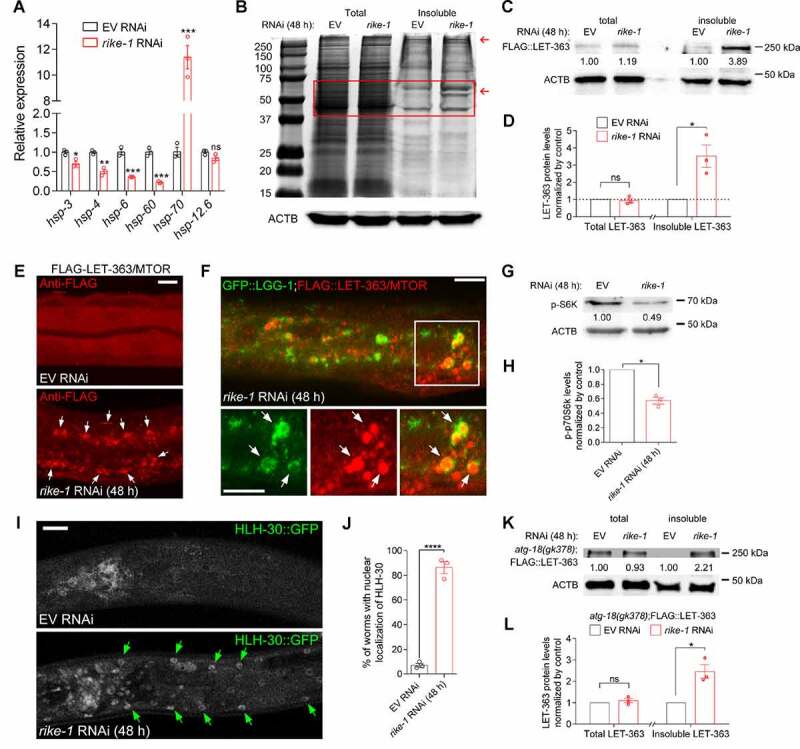
RIKE-1 loss results in proteostasis imbalance and MTOR aggregation. (A) Relative expression of mitochondrial chaperones (*hsp-6* and *hsp-60*), ER chaperones (*hsp-3* and *hsp-4*), and a cytosolic chaperone (*hsp-70*) in *rike-1* RNAi (48 h) larvae. *N* = 3 independent biological replicates. ns, *P* = 0.0822. (B) Staining of total and detergent-insoluble proteins of control and *rike-1* RNAi (48 h) worm extracts using Coomassie Brilliant Blue. Red arrows indicate the bands containing increased levels of insoluble proteins compared to the control bands. (C and D) Western blot analysis of FLAG::LET-363 in total and insoluble protein extracts of control and *rike-1* RNAi (48 h) larvae using anti-FLAG antibodies. The band intensities, quantified by ImageJ, are indicated. The levels of FLAG-LET-363 in total and insoluble protein extracts of control larvae (normalized by actin levels) are separately set to 1.00. Quantification of protein levels (*n* = 3 independent biological replicates; ns, *P* = 0.6568) is shown in D. (E) Immunostaining of FLAG::LET-363 in control and *rike-1* RNAi (48 h) larvae, showing accumulation of FLAG::LET-363 aggregates (arrows) in *rike-1* RNAi intestine. Three biologically independent RNAi experiments were performed with similar results (*n* = 10 animals). Scale bar: 10 μm. (F) Colocalization of autophagosomes labeled by GFP::LGG-1 with the accumulated FLAG::LET-363 aggregates (arrows) in *rike-1* RNAi (48 h) larvae. Scale bars: 10 μm. (G and H) Western blot analysis of phosphorylated S6K in protein extracts of control and *rike-1* RNAi (48 h) larvae. The level of p-S6K in control larvae (normalized by actin levels) is set to 1.00. Quantification of p-S6K levels (*n* = 3 independent biological replicates) is shown in h. (I and J) *rike-1* RNAi promotes the nuclear localization of HLH-30::GFP (I); quantification is in (J) (*n* = 3 independent biological replicates, about 20 animals were examined for each replicate). Arrows indicate nuclear HLH-30::GFP signals. Scale bars: 10 μm. (K and L) *atg-18(gk378)* mutation does not suppress FLAG::LET-363 aggregation induced by *rike-1* RNAi, as evidenced by western blotting and protein level quantification (*n* = 3 independent biological replicates). All statistical analyses were performed using two-tailed unpaired *t*-tests. Error bars indicate mean ± SEM. *P < 0.05, ***P < 0.001, ****P < 0.0001. ns, not significant.

Activation of autophagy and enlargement of lysosomes are hallmarks of proteotoxic stress. To explore the origin of the stress, we examined the expression of several heat shock protein (HSP) genes. The mRNA level of the cytosolic unfolded protein response gene *hsp-70* was increased about 10-fold in *rike-1* RNAi animals compared to empty vector control animals, whereas the mRNA levels of an ER chaperone (*hsp-3* and *hsp-4*) and mitochondrial chaperones (*hsp-6* and *hsp-60*) were not increased (Figure 6A). Taken together, these results indicated that *rike-1* knockdown appears to disrupt proteostasis in the cytoplasm, consequently stimulating autophagy. Imbalanced proteostasis is often accompanied with widespread protein aggregation. To determine whether this is also the case in *rike-1* knockdown animals, we extracted insoluble proteins at the early stage of *rike-1* RNAi (48 h), a time point when autophagy has been induced. Intriguingly, we found that, compared to control animals, the insoluble protein fractions in *rike-1* RNAi animals were significantly increased while total protein levels remained constant (Figure 6B). Given that the MTOR complex is the master regulator of autophagy [12] and that LET-363/MTOR deficiency in *C. elegans* causes larval arrest and intestinal atrophy similar to *rike-1*-deficient worms [49], we specifically examined whether the LET-363/MTOR formed protein aggregates. Indeed, in *rike-1*-deficient worms, the LET-363 level was significantly higher in the insoluble fraction, yet was almost unaltered in total protein (Figure 6C,D). LET-363 aggregation upon *rike-1* knockdown was also observed by immunofluorescence analysis (Figure 6E). Furthermore, LET-363 aggregates were enclosed in GFP::LGG-1 autophagosomes, implying an eventual degradation of LET-363 aggregates (Figure 6F). Next, we assessed the level of the LET-363/MTOR substrate p-RSKS-1/RPS6KB1/p70 S6K, and found that *rike-1* RNAi resulted in a decreased p-RSKS-1 level (Figure 6G,H), in line with decreased LET-363/MTOR activity derived from MTOR sequestration. Additionally, we investigated the localization of another direct target of MTOR, HLH-30/TFEB, which can translocate to the nucleus to activate autophagy-related genes in response to inhibition of MTOR signaling [50]. As expected, *rike-1* RNAi resulted in significant nuclear translocation of HLH-30::GFP (Figure 6I,J). To determine whether LET-363 aggregation preceded hyperactivated autophagy in *rike-1*-deficient worms or *vice versa*, we crossed FLAG-LET-363 into *atg-18(gk378)* mutants. We found that FLAG-LET-363 aggregation was not suppressed in *atg-18(gk378);rike-1(RNAi)* animals (Figure 6K,L), which indicates that autophagy upregulation is one of the downstream consequences of proteotoxic stress. These findings suggest that RIKE-1 loss inhibits the MTOR signaling pathway to activate autophagy, at least in part, through inducing MTOR complex aggregation.

### Reduction of Insulin/IGF-1 signaling mitigates MTOR aggregation and autophagy overactivation

**Figure 7.**
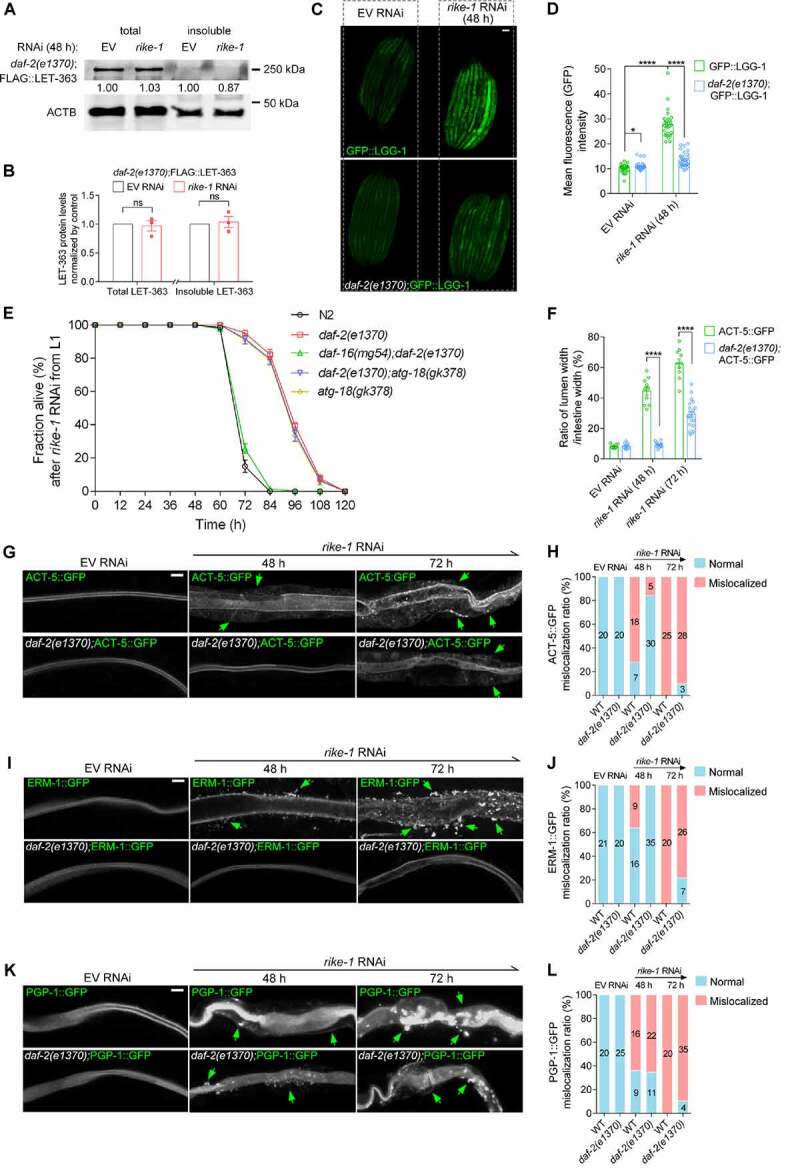
Reduction of Insulin/IGF-1 signaling mitigates MTOR aggregation and autophagy overactivation. (A and B) *daf-2(e1370)* mutation inhibits FLAG::LET-363 aggregation induced by *rike-1* RNAi, as evidenced by western blotting and protein level quantification (*n* = 3 independent biological replicates. ns, *P* = 0.7640, 0.7224). The levels of FLAG-LET-363 in total and insoluble protein extracts of control larvae (normalized by actin levels) are separately set to 1.00. (C and D) Highly increased expression of LGG-1::GFP induced by *rike-1* RNAi (48 h) is suppressed by *daf-2(e1370)* mutation (C); quantification of fluorescence intensity is in (D) (*n* = 29 each in LGG-1::GFP group; *n* = 33 each in *daf-2(e1370)*;LGG-1::GFP group). Scale bar: 50 μm. (E) The short lifespan of *rike-1* RNAi animals is partially suppressed to a similar extent by *daf-2(e1370) or atg-18(gk378)* single- and *daf-2(e1370);atg-18(gk378)* double- mutations, but not by *daf-2(e1370);daf-16(mg54)* double mutation. *n* = 5 independent biological replicates. All statistical analyses were performed using two-tailed unpaired *t*-tests. ****P < 0.0001. Error bars indicate mean ± SEM. (F-L) *daf-2(e1370)* mutation suppresses the mislocalization and cytoplasmic foci of ACT-5::GFP (G) and ERM-1::GFP (L), but not PGP-1::GFP (K, green arrows) caused by *rike-1* RNAi. (F) Quantification of the ratio of lumen width/intestine width shows that the enlarged intestinal width induced by *rike-1* RNAi is suppressed in *daf-2(e1370)* mutants (*n* = 10,10,10,16,10,21). Quantification of mislocalization of ACT-5::GFP (H), ERM-1::GFP (J) and PGP-1::GFP (L) in *rike-1*(*RNAi*) and *daf-2(e1370;rike-1*(*RNAi)* worms. *n* values are indicated in the figures from three biologically independent RNAi experiments. Scale bars: 10 μm. All statistical analyses were performed using two-tailed unpaired *t*-tests. Error bars indicate mean ± SEM. *P < 0.05, ***P < 0.001, ****P < 0.0001. ns, not significant.

We asked whether alleviating proteotoxic stress would rescue autophagy overactivation. Proteostasis is tightly regulated by the DAF-2 insulin/IGF-1 signaling pathway via the downstream effector DAF-16/FOXO [51]. We found that RIKE-1 loss led to accumulation of DAF-16::GFP aggregates in the intestinal cells (Figure S5A and S5D). The *atg-18(gk378)* mutation did not prevent DAF-16 from aggregating in *rike-1 RNAi* worms; rather, DAF-16 aggregation was slightly enhanced (Figure S5B and S5D). This suggests that, like LET-363 aggregates, DAF-16 aggregates were not caused by hyperactivated autophagy, but might be substrates of autophagy [52]. Abrogating DAF-2 signaling induces DAF-16 nuclear translocation to modulate stress responses [53]. Intriguingly, *daf-2(1370)* mutation significantly alleviated the aggregation of DAF-16 (Figure S5C and S5D) and LET-363 (Figure 7A,B) in *rike-1(RNAi)* worms, indicating that improving proteostasis by DAF-2-DAF-16 signaling axis prevents aggregate formation of DAF-16 and LET-363/MTOR. Although the expression of GFP::LGG-1 was slightly elevated in *daf-2* mutants as reported [54], the markedly increased expression of GFP::LGG-1 induced by *rike-1* RNAi was significantly suppressed by *daf-2(1370)* mutation (Figure 7C,D). This implies that reduced proteostasis capacity is the cause of autophagy overactivation. *daf-2(1370)* mutation also extended the lifespan of *rike-1(RNAi)* animals in a DAF-16-dependent manner. Typically, *atg-18(gk378)* mutation shortens the lifespan of *daf-2(e1370)* mutants [45]. Under the *rike-1* RNAi condition, *atg-18(gk378)* mutation neither extended nor shortened the lifespan of *daf-2(1370)* mutants (Figure 7E). This result is consistent with our notion about the detrimental role of autophagy induced by *rike-1* knockdown (see Figure 4 cytotoxicity section for details), and it also suggests that DAF-2 and ATG-18 contribute to the same process along the RIKE-1-mediated proteostasis-autophagy-trafficking axis. Furthermore, lessening proteotoxic stress through *daf-2(1370)* significantly rescues the lumen widening (Figure 7F) and mislocalization of ERM-1 and ACT-5, but not that of PGP-1, in *rike-1* RNAi animals (Figure 7G-L). Taken together, these results demonstrate that improving proteostasis by *daf-2*/InsR inhibition can suppress autophagy hyperactivation and subsequently restore the intestinal morphology of *rike-1-*deficient animals.

## Discussion

**Figure 8.**
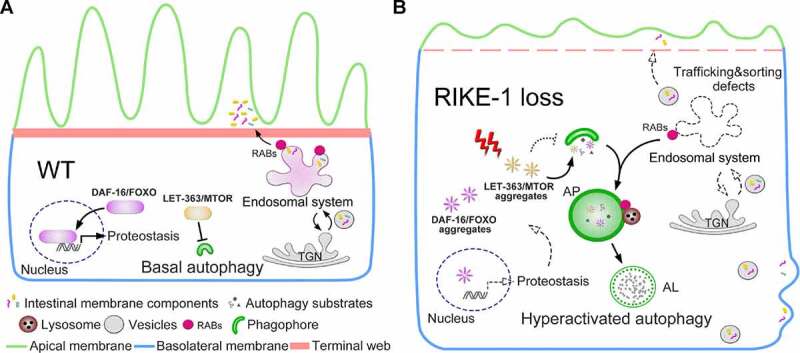
Proposed model of the role of RIKE-1 in intestinal morphogenesis. (A) In wild-type animals, the intestinal cells ensure that proteostasis stays balanced and autophagy is kept at basal level, partially through MTOR signaling and FOXO-mediated gene expression. Epithelial integrity is maintained through the coordinated and polarized trafficking of membrane-associated proteins via endosomal systems. (B) RIKE-1 loss generates proteotoxic stress and leads to sequestration of LET-363/MTOR complex and overactivation of autophagy, which in turn hijacks the endosomal trafficking system and subsequently impairs endosomal sorting and trafficking of membrane components. Proteostasis positively regulated by DAF-16/FOXO is also disrupted by *rike-1* loss via aggregation of DAF-16/FOXO.

We have demonstrated that the novel protein RIKE-1 contributes to epithelial membrane morphogenesis, at least in part, by controlling protein homeostasis. Imbalanced protein homeostasis triggers aggregation of the LET-363/MTOR complex as well as many other proteins, which subsequently overactivates autophagy, degrades endosomes and derails membrane trafficking (Figure 8). Emerging evidence suggests that protein aggregation is often part of the cellular defense against imbalanced protein homeostasis [55–58]. Similarly, autophagy helps the cell adapt to and cope with stress by eliminating misfolded proteins and damaged organelles [59–62]. Protein aggregation and autophagy upregulation are both supposed to be reversible. Paradoxically, in response to RIKE-1 loss-induced proteotoxic stress, it seems that the insoluble proteins are terminally deposited as aggregates and autophagy is constitutively induced (until the worms die). A consequence is the sequestration of MTOR into aggregates, leading to MTOR inhibition followed by autophagy upregulation. Alleviating proteotoxicity by reducing DAF-2 insulin/IGF-1 signaling prevents protein aggregation and inhibits autophagy overactivation, but not *vice versa* (Figure 7A-D and S5A-D). This demonstrates that hyperactivated autophagy indeed occurs downstream of proteotoxic stress. Excessive autophagy depletes endosomes, probably by using them for autophagosome biogenesis. Exploitation of the endosomal system compromises its roles in membrane trafficking and disrupts plasma membrane composition. The aggravated cellular stress caused by the damaged membrane may serve as an alternative explanation for the irreversible response. Taking these lines of evidence together, we envision that RIKE-1 is a major factor that shapes cellular proteostasis.

RIKE-1 contains a RING domain and Kelch repeats, and is homologous to the mammalian actin-binding protein IPP based on the similarity of its Kelch domain. Members of the Kelch repeat protein family have a known role in regulating cytoskeletal organization [30]. However, disruption of the actin cytoskeleton cannot lead to cellular stress that is comparable with the stress that occurs under *rike-1* deficiency. This suggests that the interaction of RIKE-1 with actin, if any, is only part of the story. RIKE-1 also carries a Cys3HisCys4-type Zinc finger domain at the N-terminus and this group of RING domain proteins includes many E3 ubiquitin ligases [63]. In fact, E3 ligases are implicated in both proteome balance and epithelial morphogenesis [64]. Further work is required to elucidate the detailed molecular mechanism of how RIKE-1 controls protein homeostasis by preventing the accumulation of surplus and aberrant protein species.

## Materials and methods

### Strains and alleles

The *C. elegans* strains were maintained and cultured on nematode growth medium (NGM; 3 g/L NaCl [Sigma-Aldrich, S7653], 2.5 g/L peptone [Thermo Fisher Scientific, 211677], 17 g/L agar [Sigma-Aldrich, A6686], 25 mM K_2_HPO_4_/KH_2_PO_4_ [Sigma-Aldrich, P3786, P0662; pH 6.0], 1 mM CaCl_2_ [Sigma-Aldrich, C1016], 1 mM MgSO_4_ [Sigma-Aldrich, M7506], 5 mg/L cholesterol [Sigma-Aldrich, C8667]) plates seeded with OP50 *E. coli* using standard techniques [65] at 20°C, with the exception of temperature-sensitive *daf-2(e1370)* mutants, which were grown at 16°C. All the strains used in this study are listed in Table S2.

### RNAi knockdown experiments

RNA interference (RNAi) experiments were performed by the feeding method as previously described [43]. For standard RNAi, about 10 L4 larvae were picked and cultured on RNAi plates (NGM containing 2 mM IPTG [Sigma-Aldrich, I6758] and 50 μg/ml carbenicillin [Sigma-Aldrich, C3416]) seeded with bacterial clones of target genes and F1 progeny larvae were examined after 72 h. Standard *rike-1* RNAi leads to fully penetrant L1 lethality. We therefore also performed mild RNAi, starting from the L1 stage, for better evaluation of changes in physiological processes of *rike-1*-deficient larvae. Briefly, eggs from over 50 bleached adults were allowed to hatch on *rike-1* RNAi plates. After 48 h, most of the worms developed into L3-L4 larvae and were scored for early phenotypes. At 72 h, most of the larvae were arrested at the L3-L4 stages and scored for late phenotypes. Even milder RNAi conditions can be achieved by mixing the *rike-1* RNAi clone with the RNAi bacteria containing the L4440 vector plasmid (Addgene, 1654; Andrew Fire) at different proportions when necessary. Worms were fed with RNAi bacteria containing the L4440 empty vector plasmid as a control treatment (EV RNAi). All RNAi clones were confirmed by sequencing.

### Gene editing by CRISPR-Cas9

The mutant *rike-1(g55t;c72a)* carries two premature stop codons in close proximity (E19X,C24X). Design of CRISPR-Cas9 plasmids to create this mutant was performed as previously described [66]. The 20 bp gRNAs for creating DNA double-strand breaks in the *rike-1* genomic locus were cloned into pPD162 Peft-3::Cas9;PU6::empty sgRNA vector (Addgene, 47549; Bob Goldstein). An exogenous donor oligonucleotide for homologous repair was designed and synthesized, including ~50 bp of flanking homology on either side and additional synonymous mutations to ablate the gRNA cleavage site and introduce an FspI restriction site for convenient screening. Fifty ng/μl gRNAs and 500 nM donor oligonucleotides were used for injection. A plasmid containing gRNA and repair oligonucleotide for the *dpy-10(cn64)* mutation, which confers a dominant rolling phenotype, was co-injected at a concentration of 50 ng/μl as a screening marker [40]. Roller animals screened from F1 progeny were singled and, after laying eggs, were picked out for single-worm PCR and restriction enzyme digestion for validation of the *rike-1(g55t;c72a)* mutation. Non-roller F2 heterozygotes were singled and screened for homozygosity. After confirming that *rike-1(g55t;c72a)* mutation causes lethality, *rike-1(g55t;c72a)* mutants and the *rike-1(g55t;c72a)* derivatives were maintained as heterozygotes. To test heterozygotes, 10–20 animals were singled out for each generation, and the plates containing dead worms were picked for phenotypic scoring and genotyping. For genotyping, the arrested animals were used as templates for single-worm PCR followed by sequencing.

Similarly, to generate the knockout mutant *rike-1(syb1165)*, two pairs of gRNAs were inserted into Cas9-sgRNA plasmids and co-injected with pRF4 *rol-6(su1006)* [67] as an injection marker at a concentration of 50 ng/μl each. Roller F1 progeny were isolated, and their progeny were examined by PCR. The *rike-1(syb1165)* knockout mutation was generated and balanced by SunyBiotech (China). To generate the *rike-1(syb1165)* mutants carrying GFP::LGG-1 and ACT-5::GFP, *rike-1(syb1165)* mutants were crossed with the reporter strains followed by crossing back with the nT1 balancer. The specificity of mutations was confirmed by sequencing. The sequences of gRNAs and repair oligonucleotides are provided in Table S3.

### Bafilomycin A_1_ treatment

To examine the autophagic flux, GFP::LGG-1-expressing worms treated with mild RNAi for 48 h were picked out and incubated in M9 (3 g/L KH_2_PO_4_, 6 g/L Na_2_HPO_4_ [Sigma-Aldrich, S3264], 5 g/L NaCl, 1 mM MgSO_4_) containing 25 mM bafilomycin A_1_ (Sigma-Aldrich, B1793) or DMSO for 6 h before imaging.

### Quantitative real-time PCR (qRT-PCR)

Total RNA was extracted using TRIzol reagent (TaKaRa, 9108) following the standard protocol. RNA was quantified based on its OD at a wavelength of 260 nm and RNA purity was assessed by the absorbance ratios of 260:280 and 260:230. Reverse transcription was performed using a PrimeScript^TM^ RT Reagent Kit (TaKaRa, RR047A). Quantitative RT-PCR was performed using Fast SYBR Green Master Mix (Thermo Fisher Scientific, 4385616) and the ABI7500 Fast Real-Time PCR Detection System. Relative mRNA expression levels were normalized to *tba-1* and analyzed using the ΔΔC_t_ method. Primers used for the RT-PCR experiments are listed in Table S3.

### Immunostaining

The immunofluorescence staining of worms was performed as previously described [43]. Briefly, the collected worms were permeabilized by flash-freezing in liquid nitrogen. Fixation was carried out by sequential incubation in methanol and acetone at −20°C. The worms were blocked in PBS buffer (Sigma-Aldrich, P4417) containing 5% BSA (Sigma-Aldrich, SLCF3318) and 0.5% Triton X-100 (Sigma-Aldrich, 9036–19-5), then incubated with primary antibodies at 4°C overnight, followed by a series of incubation and washing steps in PBST (0.05% Tween 20 [Sigma-Aldrich, P9416]) buffer. The worms were incubated with secondary antibodies for 1 h, then extensively washed and mounted with anti-fade Fluorescence Mounting Medium (Sigma-Aldrich, F6057). Primary and secondary antibodies were used at the following concentrations: anti-IFB-2 (Developmental Studies Hybridoma Bank [DSHB], 528311; 1:20); anti-DLG-1 (DSHB, 2617529; 1:10); anti-FLAG (Sigma-Aldrich, F7425; 1:50); goat anti-mouse IgG-TRITC (Sigma-Aldrich, T5393; 1:100).

### Immunoblotting

To extract proteins, worms were lysed in RIPA buffer (Millipore, 20–188) supplemented with protease inhibitor mixture (Roche, 11836170001), then mildly sonicated and centrifuged to remove worm residues. After quantification by BCA assay (Thermo Fisher Scientific, 23227), samples containing equal amounts of protein were resolved on 12% sodium dodecyl sulfate-polyacrylamide gel electrophoresis (SDS-PAGE) gels with running buffer (0.025 M Tris–HCl, pH 8.3, 0.192 M glycine, 0.1% SDS) and then transferred to polyvinylidene fluoride membranes (Bio-Rad, 6116) at 70 V for 90 min. Membranes were sequentially incubated with primary- and HRP-conjugated secondary antibodies, then developed with Clarity™ western ECL substrate (Bio-Rad, 1705061) and imaged using ChemiDoc™ Imaging Systems (Bio-Rad). The blot intensity was quantified by ImageJ software. The following primary antibodies were used: Mouse anti-GFP (Roche, 11814460001; 1:2000); rabbit anti-mCherry (Proteintech, 26765-1-AP; 1:2000); rabbit anti-phospho-RPS6KB/p70 S6 kinase (Thr398) (Cell Signaling Technology, 9209; 1:1000); mouse anti-ACTB/β-actin (C4) (Santa Cruz Biotechnology, sc-47778; 1:2000); mouse anti-FLAG M2-peroxidase (Sigma-Aldrich, A8592; 1:2000); rat anti-CPL-1 (a gift from Xiaochen Wang lab; Institute of Biophysics, Chinese Academy of Sciences; 1:1000). The following secondary antibodies were used: Goat anti-mouse IgG-peroxidase (Sigma-Aldrich, A5278; 1:2000); goat anti-rat IgG-peroxidase (Abcam, ab97057; 1:2000); goat anti-rabbit IgG-peroxidase (Abcam, ab6721; 1:2000).

### Extraction of insoluble proteins

Insoluble proteins were extracted from worm lysates as previously described [68] with some modifications. Briefly, worms were lysed with RIPA buffer supplemented with protease inhibitor. After mild sonication for 15 min at 4°C, worm lysates were centrifuged at 400 g for 5 min to remove debris. To serve as total protein,100 µl of the protein suspension was saved and mixed with 2X urea-SDS buffer (2% SDS, 50 mM dithiothreitol, 50 mM Tris, pH 8.0, 16 M urea [Sigma-Aldrich, U5378]) at RT. The rest was centrifuged at 21,000 g for 20 min at 4°C to remove detergent-soluble protein fractions. The pellet was resuspended in 75 μl urea-SDS buffer (8 M urea) at RT and used as the insoluble protein fraction. Both total and insoluble protein samples were kept at −80°C for later examination or resolved on a 10% SDS-PAGE gel followed by Coomassie Brilliant Blue (Bio-Rad, 1610436) staining.

### Transmission electron microscopy (TEM) analysis

For TEM analysis, 10–30 living L3-L4 larvae from control and *rike-1(RNAi)* groups were picked into a 200-μm specimen carrier filled with *E. coli* for rapid freezing in a high-pressure freeze (HPF) device (Leica HPM100, Germany). Following HPF, the fast-frozen samples were immersed into a cryovial containing 2% osmium tetroxide in 98% acetone/2% water for a freeze substitution (FS) procedure in an FS unit (Leica EM AFS, Germany) with the following parameter settings: T1 =  −90°C for 72 h, S1 =  5°C/h, T2 =  −60°C for 12 h, S2 =  5°C/h, T3 =  −30°C for 10 h, followed by a slow warming procedure to 10°C (5°C/h). After completion of FS, samples were rinsed extensively with acetone, then stained with 0.5% uranyl acetate dissolved in 90% acetone/10% methanol for 2 h in the dark at RT. After another series of extensive rinses with acetone, the samples were successively infiltrated with a mixture of resin (Electron Microscopy Sciences, EMbed 812) and acetone at ratios of 1:2, 1:1 and 3:1 at RT for 5 h, overnight, and 12 h, respectively. To ensure complete infiltration, the worms underwent 3–4 additional changes with 100% resin over the next five days with a tissue rotator. In 100% resin, the worm plus *E. coli* can easily come out of the holder; therefore, the worms were repositioned for the convenience of cross sectioning using a stereoscope in an embedding mold with fresh resin containing 1.5% benzyldimethylamine (Sigma-Aldrich, 185582). The block was allowed to polymerize at 60°C for 48 h before being trimmed and sectioned on an ultramicrotome (UC6, Leica Biosystem, Germany) with diamond knives (Diatome, Switzerland). Serial sections of worm samples were automatically collected using the Auto CUTS device and were observed with a JEM-1400 (JEOL) operating at 80 kV.

### Fluorescence microscopy

Live worms were paralyzed in 5 μl 10 mM sodium azide (Sigma-Aldrich, S2002) on glass slides and observed directly under a Zeiss LSM710 confocal microscope (Carl Zeiss MicroImaging) equipped with a Plan-Apochromat DIC 63x/1.4 oil objective. For comparison of fluorescence intensity in Figure 6(b,c), the paralyzed worms were mounted in order on 2% agarose pads for direct detection using a LSM710 microscope with a 10x/0.3 dry objective. Single plane images were taken at 0.2 μm or smaller intervals along the *z* axis and maximum intensity projection images were generated by integrating 6–10 sections of single plane images. Multi-channel images were acquired by sequential scanning of individual channels to eliminate bleed-through between different channels. To distinguish the signals of interest from intestinal autofluorescence, the DAPI channel (405 nm excitation) was used to identify autofluorescent granules [44]. The images were processed using Adobe Photoshop CS6.

### Statistical analysis

GraphPad Prism 6 software was used to perform statistical tests and generate graphs. Statistical differences were determined by two-tailed unpaired Student’s *t*-test and bar graphs are shown as mean ± standard error of the mean (SEM). A *P* value less than 0.05 was considered statistically significant. *P < 0.05; **P < 0.01; ***P < 0.001; ****P < 0.0001; ns, no significance. Statistical parameters including the definitions, exact values of *n*, and statistical significance are indicated in the figures and figure legends. All experiments were independently repeated at least twice with similar results. The density of immunoblot bands and fluorescence intensity of microscopy images were quantified using ImageJ software.

## Supplementary Material

Supplemental Material
