# Supplementary material for: Proteotoxic stress disrupts epithelial integrity by inducing MTOR sequestration and autophagy overactivation
Source: Autophagy. 2022 May 6;19(1):241–55. doi: 10.1080/15548627.2022.2071381 (PMC9809964; doi:10.1080/15548627.2022.2071381)
Supplement: Supplemental Material [file KAUP_A_2071381_SM3866.zip › Supplemental Figures and Tables R3.docx]

**Supplementary Figure legends**

**
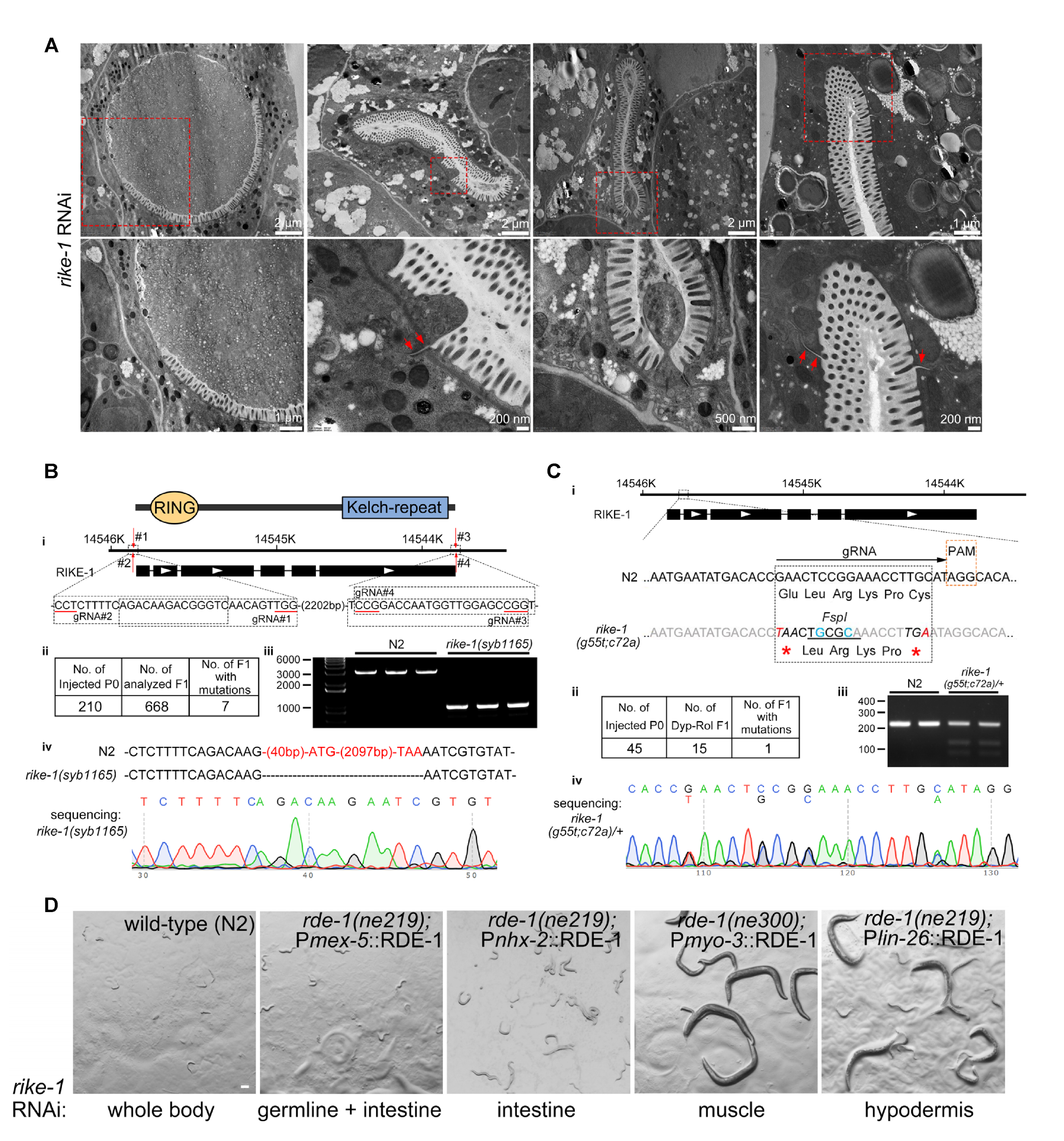
**

**Figure S1.** RIKE-1 is required for intestinal development. (**A**) TEM micrographs of intestinal cross-sections of *rike-1* RNAi larvae showing various aspects of disruption, ranging from loss of microvilli (left) to apical disorganization and membrane rupture (middle), and leaky membrane (right, red arrows) due to disruption of the terminal web. High-magnification views of the boxed regions are shown in the lower panels. (**B**) Schematic representation of RIKE-1 protein containing an N-terminal RING domain and a C-terminal Kelch-repeat domain. The *rike-1(syb1165)* mutation that deletes the whole gene was generated using CRISPR/Cas9 (i,ii), confirmed by PCR (iii) and sequencing (iv). (**C**) The *rike-1(g55t;c72a)* mutation that carries two premature stop codons was generated using CRISPR/Cas9 (i,ii), confirmed by PCR (iii) and sequencing (iv). (**D**) Tissue-specific RNAi confirms the requirement for RIKE-1 in intestinal development. Scale bar: 100 μm.

**
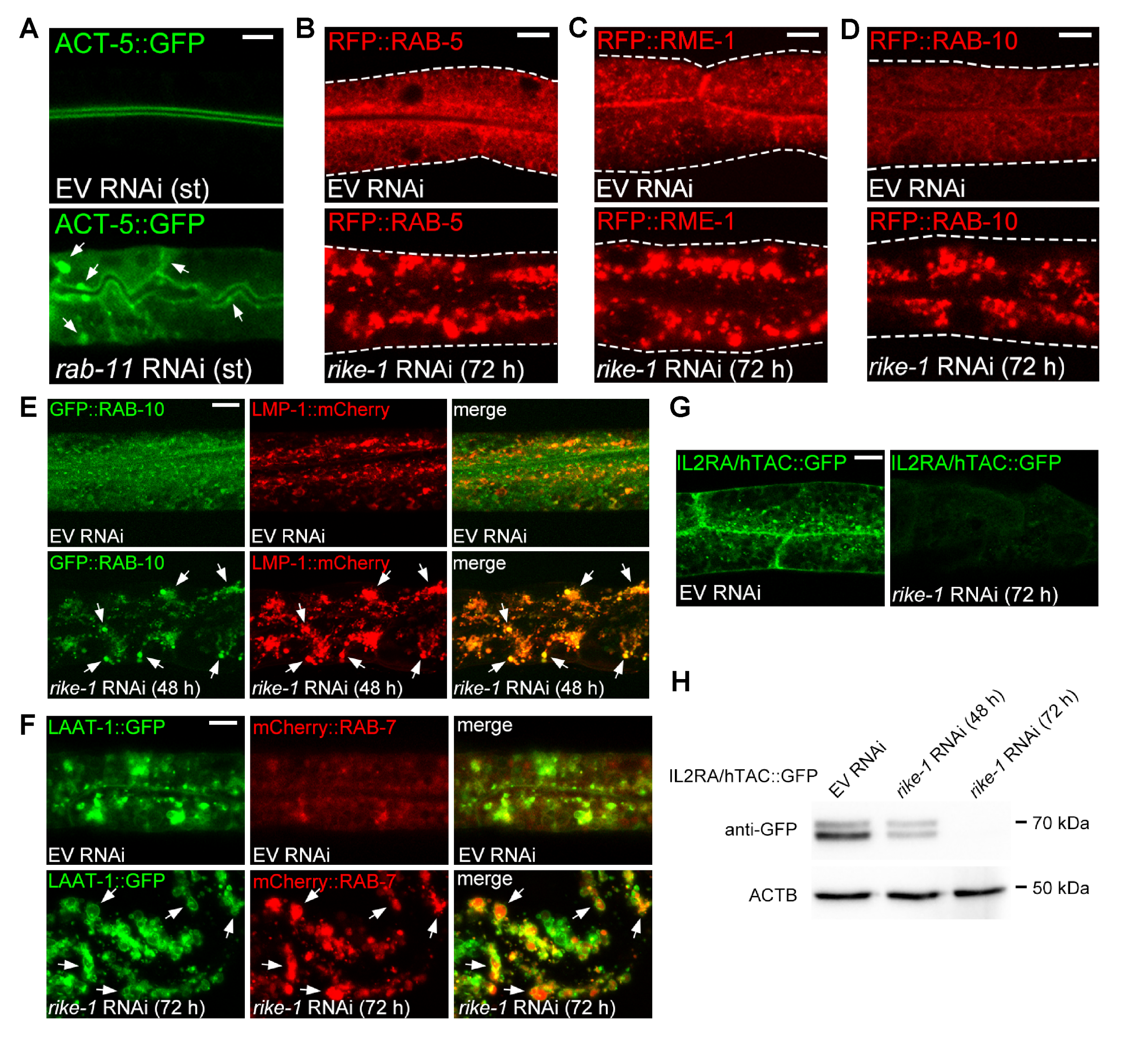
**

**Figure S2.** RIKE-1 loss causes enlargement, clustering and excessive degradation of endosomes. (**A**) RNAi knockdown of the recycling endosome marker RAB-11 leads to basolateral displacement (arrowheads) and cytoplasmic accumulation (arrows) of ACT-5::GFP. Three biologically independent RNAi experiments were performed with similar results (*n*=10 animals). (**B**-**D**) Compared with control, *rike-1* RNAi leads to dramatic enlargement and clustering of endosomes tagged with RFP, including RAB-5-positive early endosomes (**B**), RME-1-positive recycling endosomes (**C**) and RAB-10-positive recycling endosomes (**D**) at 72 h. (**E** and **F**) Colocalization of endosomes with lysosomes. In *rike-1*-deficient worms, enlarged recycling endosomes (positive for GFP::RAB-10) are colocalized with LMP-1::mCherry-labeled lysosomes (**E**, arrows) and enlarged late endosomes (positive for mCherry::RAB-7) are enclosed in LAAT-1::GFP-labeled lysosomes (**F**, arrows). (**G** and **H**) The recycling cargo IL2RA/hTAC::GFP decreases significantly in *rike-1* RNAi animals (**G**). Western blot of IL2RA/hTAC::GFP in control and *rike-1* RNAi group is shown in (**H**). For all panels, three biologically independent RNAi experiments were performed with similar results (*n*>20 animals). Scale bars: 10 μm.

**
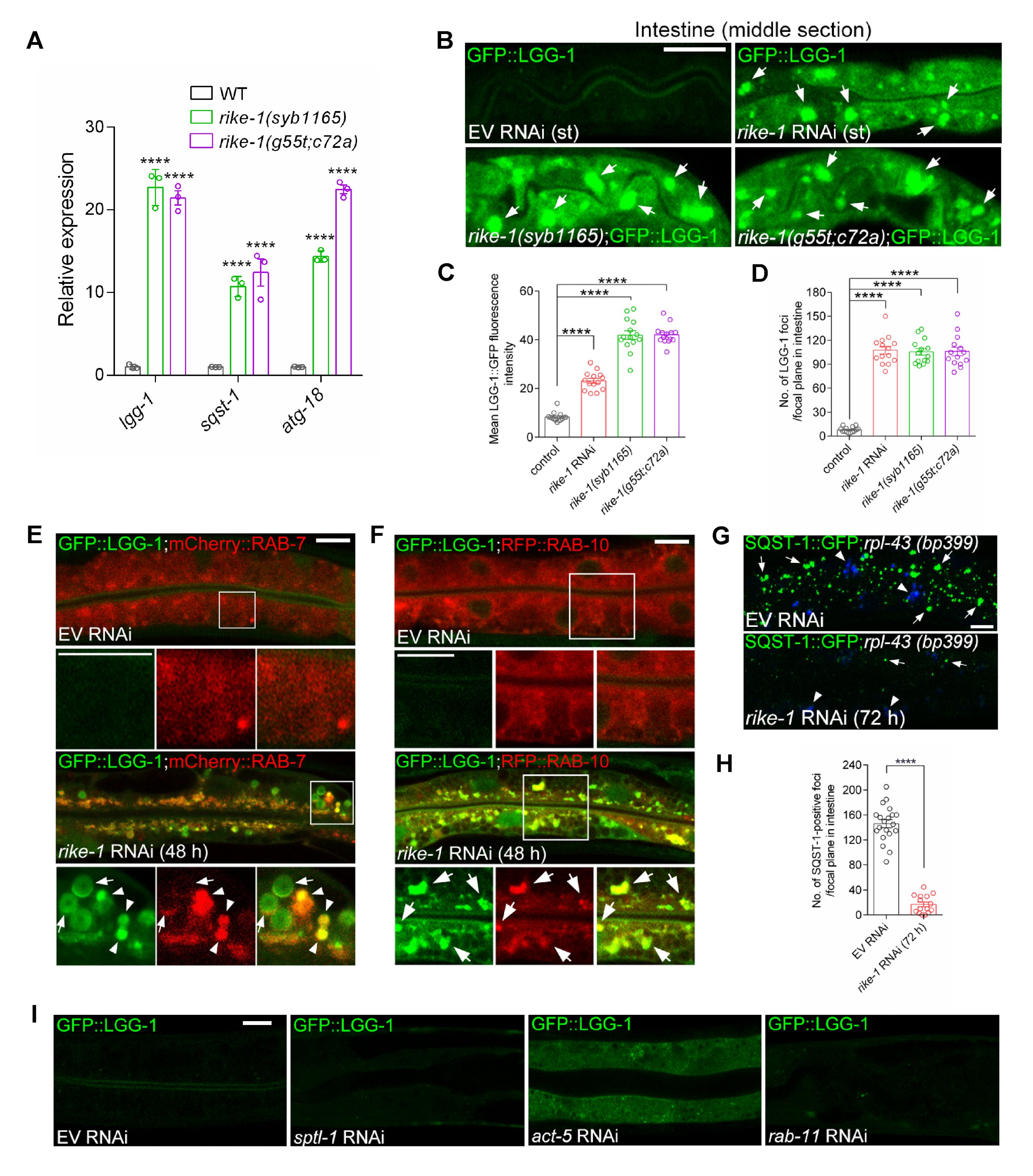
**

**Figure S3.** RIKE-1 loss leads to autophagy overactivation, which is not a secondary result of lethality or a general membrane trafficking defect.(**A**) Relative expression of *lgg-1*, *sqst-1* and *atg-18* measured by qRT–PCR in *rike-1* loss-of-function mutants versus controls. The mRNA levels were normalized to *tba-1*. *n*=3 independent biological replicates. (**B**-**D**) Confirmation of autophagy upregulation using the autophagosome reporter GFP::LGG-1 in *rike-1* RNAi, *rike-1(syb1165)* mutants and *rike-1(g55t;c72a)* mutants versus control. Quantification of fluorescence intensity and the number of GFP puncta (arrows) is shown in (**C**) and (**D**) (*n*=14 each). (**E** and **F**) Colocalization of autophagosomes labeled by LGG-1::GFP with late endosomes marked by mCherry::RAB-7 (**E**) and recycling endosomes marked by RFP::RAB-10 (**F**) in control and *rike-1* RNAi (48 h) larvae. Arrows, colocalized puncta. (**G** and **H**) *rike-1* RNAi reduces SQST-1::GFP puncta in *rpl-43(bp399)* mutants (**G**); quantification is shown in (**H**) (*n*=20,15). (**I**) Expression of LGG-1::GFP in *sptl-1(RNAi)*, *act-5(RNAi)* and *rab-11(RNAi)* animals, implying that LGG-1::GFP upregulation is not secondary to endosomal trafficking and intestinal morphology defects or lethality. Scale bar: 10 μm. All statistical analyses were performed using two-tailed unpaired *t*-tests. Error bars indicate mean ± SEM. *****P*<0.0001.


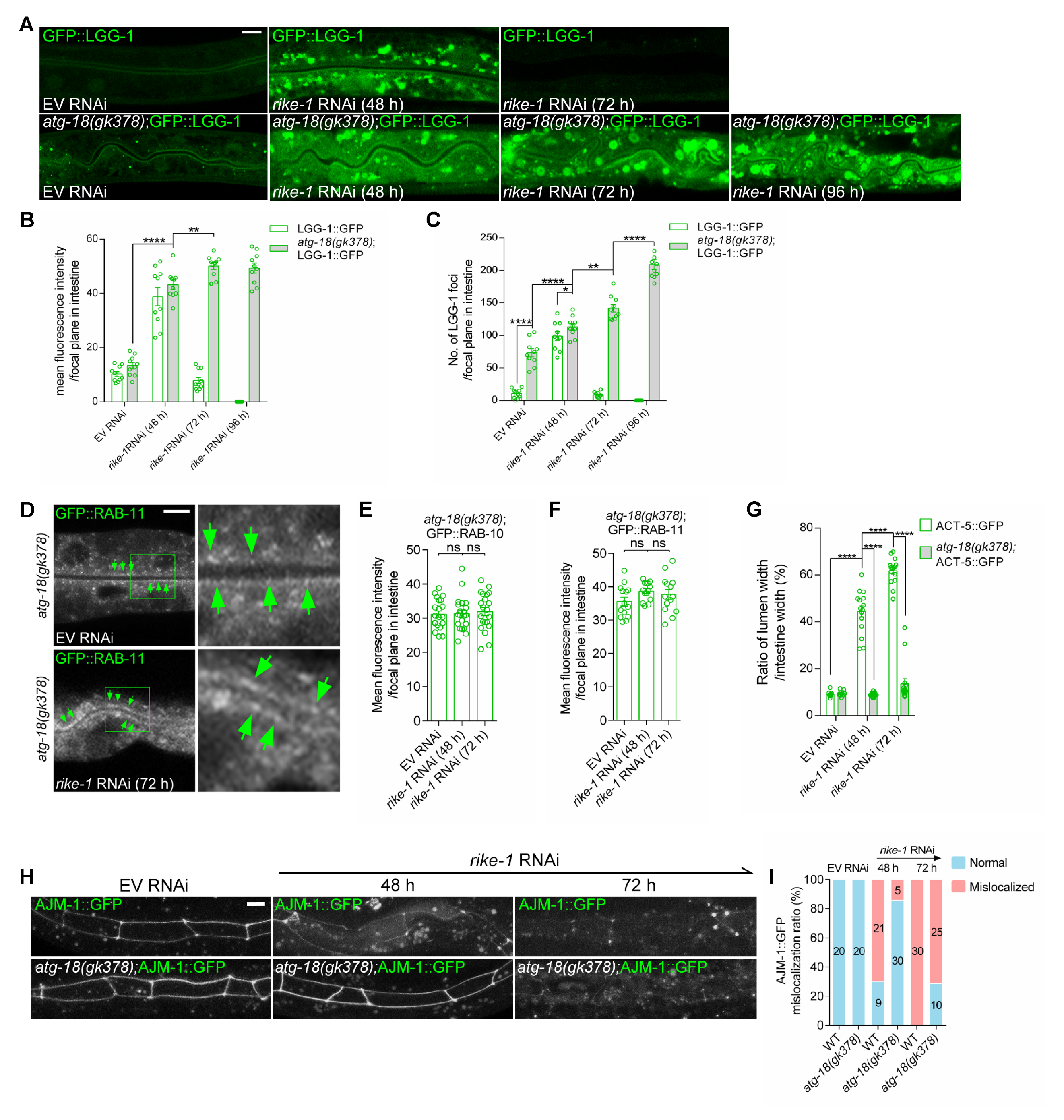


**Figure S4.** Blocking autophagy partially rescues endosomal trafficking and intestinal morphogenesis defects in *rike-1(RNAi)* worms. (**A**-**C**) *atg-18(gk378)* mutation prevents the degradation of LGG-1::GFP puncta in *rike-1* RNAi larvae (**A**). Quantification of fluorescence intensity and number of LGG-1::GFP foci is shown in (**B**) and (**C**) (*n*=10 each). (**D**) *atg-18(gk378)* mutation suppresses RAB-11::GFP clustering and degradation induced by *rike-1* RNAi (see Figure 2E for comparison). (**E** and **F**) Quantification of the fluorescence intensity of the recycling endosome marker RAB-10::GFP (**E**, see Figure S2**E** for comparison, *n*=14 each; ns, *P*=0.1208, 0.2838) and RAB-11::GFP (**F**, see Figure 2**A** for comparison, *n*=21 each; ns, *P*=0.9073, 0.6230) in *atg-18(gk378)* mutants. (**G**) Quantification of the ratio of lumen width/intestine width shows that the enlarged lumenal width induced by *rike-1* RNAi is restored in *atg-18(gk378)* mutants (*n*=10 for all EV RNAi groups; *n*=15 animals for all *rike-1* RNAi groups). (**H** and **I**) Displacement of AJM-1::GFP from apicolateral junctures caused by *rike-1* RNAi is partially alleviated in *atg-18(gk378)* mutants (**H**). Quantification of worms with clear junctional AJM-1::GFP in (**I**) (*n* values are indicated in the figure from three biologically independent RNAi experiments). 10 μm for all others. All statistical analyses were performed using two-tailed unpaired *t*-tests. Error bars indicate mean ± SEM. **P*<0.05, ***P*<0.01, ****P*<0.001, *****P*<0.0001. ns, not significant.


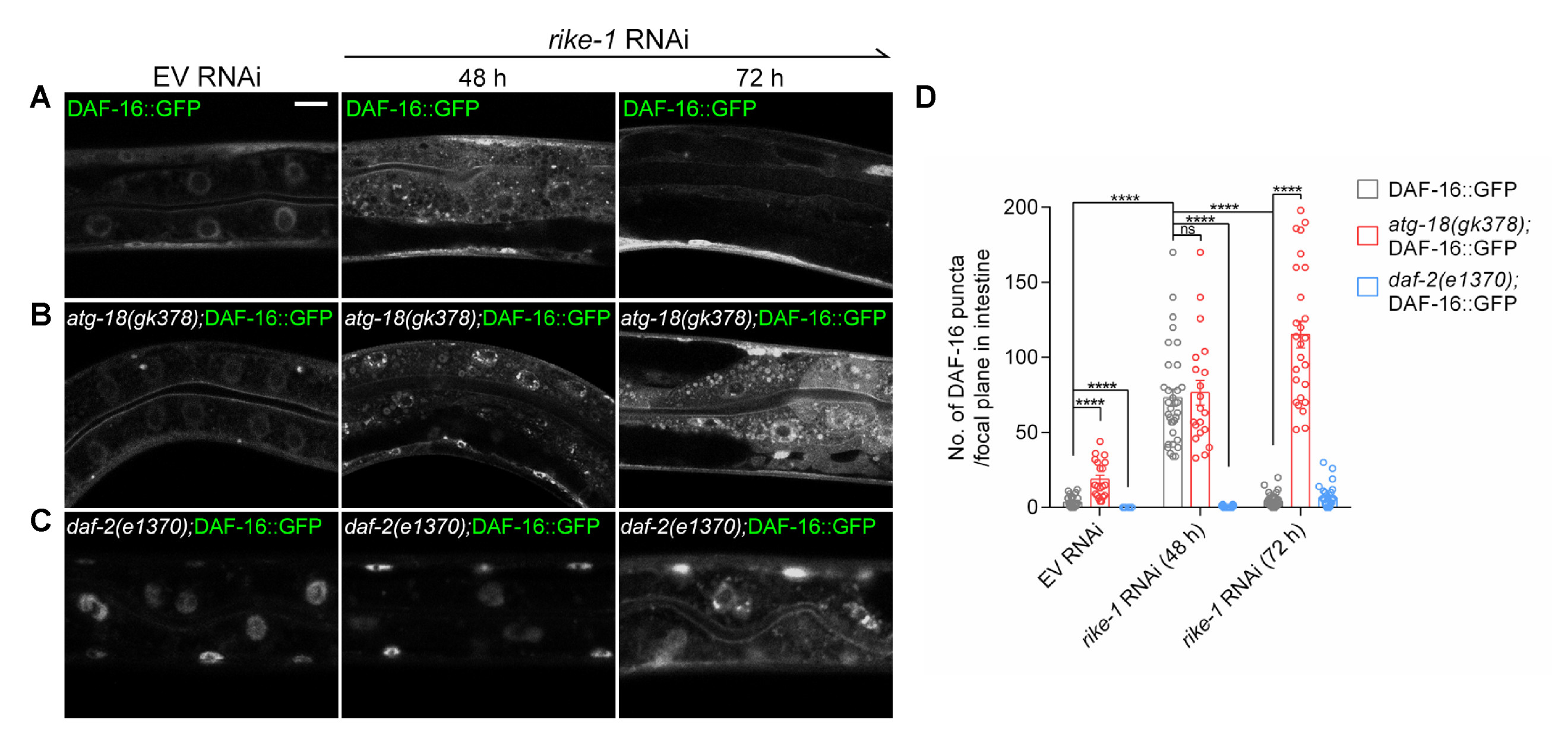


**Figure S5.** RIKE-1 loss leads to DAF-16 aggregation, which can be partially restored by reducing DAF-2 activity, not by autophagy inhibition. (**A**-**D**) Effects of *rike-1* RNAi on the expression of DAF-16::GFP in WT, *atg-18(gk378)* mutants and *daf-2(e1370)* mutants. Intestinal DAF-16::GFP aggregates increase in *rike-1*(*RNAi, 48 h*) animals, and then decline in *rike-1*(*RNAi, 72 h*) worms (**A**). Intestinal DAF-16::GFP aggregates increase in *atg-18(gk378);rike-1*(*RNAi, 48 h*) mutants, and persist and accumulate in *atg-18(gk378);rike-1*(*RNAi, 72 h*) mutants (**B**). *daf-2(e1370)* mutation delays the aggregation of intestinal DAF-16::GFP in *rike-1* RNAi animals (**C**). Quantification of intestinal DAF-16::GFP puncta is shown in (**D**). For DAF-16::GFP group, *n*=28,33,38; for *atg-18(gk378)*;DAF-16::GFP group, *n*=20,20,26; for *daf-2(e1370)*;DAF-16::GFP group, *n*=10,29,32. ns, *P*=0.7228. Scale bars: 10 μm for whole pictures, 5 μm for insets. All statistical analyses were performed using two-tailed unpaired *t*-tests. Error bars indicate mean ± SEM. *****P*<0.0001. ns, not significant.

**Supplementary Tables**

**Table S1.** RNAi screening of 11 Kelch repeat proteins in *C. elegans* using ACT-5::GFP.

| Gene | F1 progeny | Phenotypes of ACT-5::GFP in F1 larvae |
| --- | --- | --- |
| *C53A5.6* | L1 arrest (100%) | Mislocalization (100%) |
| *C53A5.9* | L4 and young adults | Superficially wild type |
| *C53A5.11* | L4 and young adults | Superficially wild type |
| *F47D12.7* | L4 and young adults | Superficially wild type |
| *ivns-1* | L4 and young adults | Superficially wild type |
| *kel-1* | L4 and young adults | Superficially wild type |
| *kel-3* | L4 and young adults | Superficially wild type |
| *kel-8* | L4 and young adults | Superficially wild type |
| *kel-10* | Embryonic lethal (1%); L4 and young adults (99%) | Superficially wild type |
| *kel-20* | Embryonic lethal (1%); L4 and young adults (99%) | Superficially wild type |
| *spe-26* | Embryonic lethal/newly hatched L1 arrest (2%); L4 and young adults (98%) | Dramatically enlarged lumen (1%) |

^L4 larvae (F0) were fed with RNAi clones, and after removing F0 larvae at 24 h, F1 progeny larvae were examined at 72 h.^

**Table S2.** Strains used in this study.

| Strain Name | Description | Source |
| --- | --- | --- |
| Wild type | N2 Bristol | CGC |
| GK70 | *unc-119(ed3); dkIs37[pact-5::GFP::pgp-1 unc-119(+)]* | CGC |
| KWN246 | *pha-1(e2123ts); Ex[popt-2::opt-2::GFP pha-1(+)]* | CGC |
| SU93 | *jcIs1[ajm-1::GFP unc-29(+) rol-6(su1006)]IV.* | CGC |
| AMJ345 | *jamSi2[mex-5p::rde-1(+)]II; rde-1(ne219)V* | CGC |
| [VP303](https://cgc.umn.edu/strain/VP303) | *rde-1(ne219)V; kbIs7 [nhx-2p::rde-1 rol-6(su1006)].* | CGC |
| WM118 | *rde-1(ne300)V; neIs9 [myo-3::HA::rde-1 rol-6(su1006)].* | CGC |
| NR321 | *rde-1(ne219)V; kzEx321[pKK1253(lin-26p::rde-1) rol-6(su1006)].* | CGC |
| RT311 | *unc-119(ed3); pwIs69[vha6p::GFP::rab-11 Cbunc-119(+)]* | CGC |
| RT476 | *unc-119(ed3); pwIs170[vha6p::GFP::rab-7 Cbunc-119(+)]* | CGC |
| RT525 | *unc-119(ed3); pwIs206[vha6p::GFP::rab-10 Cbunc-119(+)]* | CGC |
| DA2123 | *adIs2122[plgg-1::GFP::lgg-1 rol-6(su1006)]* | CGC |
| MAH215 | *sqIs11[plgg-1::mCherry::GFP::lgg-1 rol-6(su1006)]* | CGC |
| MAH235 | *sqIs19[phlh-30::hlh-30::GFP rol-6(su1006)]* | CGC |
| TJ356 | *zIs356[daf-16p::daf-16a/b::GFP rol-6(su1006)]* | CGC |
| CB1370 | *daf-2(e1370)* | CGC |
| GR1895 | *daf-2(e1370); mgIs67[daf-16p::daf-16::GFP rol-6(su1006)]* | CGC |
| GR1308 | *daf-16(mg54); daf-2(e1370)III* | CGC |
| VC893 | *atg-18(gk378)* | CGC |
| VJ266 | *fgEx12[pact-5::act-5::GFP rol-6(su1006)]* | ^1^ |
| VJ402 | *fgEx13[perm-1::erm-1::GFP rol-6(su1006)]* | ^2^ |
| RT548 | *pwIs216[pvha-6::RFP::rme-1 Cbunc-119(+)]* | ^3^ |
| FA086 | *pwIs429[pvha-6::mCherry::rab-7 Cbunc-119(+)]* | ^4^ |
| RT1087 | *pwIs414[vha-6::RFP::rab-10, Cbunc-119(+)]* | ^4^ |
| RT611 | *pwIs846[pvha-6::RFP::rab-5 Cbunc-119(+)]* | ^3^ |
| RT393 | *pwIs112[pvha-6::IL2RA/hTAC::GFP Cbrunc-119(+)]* | ^3^ |
| HZ946 | *rpl-43(bp399);bpIs151 [sqst-1p::sqst-1::GFP unc-76(+)]* | ^5^ |
| HZ4058 | *bpEx231(Plgg-1::gfp::lgg-1(g116a), pRF4(rol-6))* | ^6^ |
| HZ3340 | *bpIs322[flag::let-363]* | ^7^ |
| HZ2244 | *bpEx118[W07G4.5p::W07G4.5::mCherry unc-76(+)]* | Hong Zhang |
| HJZ073 | *mauEx31 [Plet-413::let-413::GFP::PP7, Prol-6::rol-6(su1006)]* | ^8^ |
| XW11282 | *qxIs520[pvha-6::laat-1::GFP Cbunc-119(+)]* | ^9^ |
| XW5331 | *lgg-1(bp500)* | ^9^ |
| XW5399 | *qxIs257[Pced-1::nuc-1::cherry+unc-76]* | ^9^ |
| HJZ101 | *rike-1(g55t;c72a)/+* | This study |
| HJZ102 | *rike-1(syb1165)/nT1* | This study (SunyBiotech) |
| HJZ103 | *rike-1(g55t;c72a)/+;fgEx12[pact-5::act-5::GFP rol-6(su1006)]* | This study |
| HJZ104 | *rike-1(syb1165)/nT1;fgEx12[pact-5::act-5::GFP rol-6(su1006)]* | This study |
| HJZ105 | *rike-1(g55t;c72a)/+;adIs2122[plgg-1::GFP::lgg-1 rol-6(su1006)]* | This study |
| HJZ106 | *rike-1(syb1165)/nT1;adIs2122[plgg-1::GFP::lgg-1 rol-6(su1006)]* | This study |
| HJZ107 | *pwIs206[vha6p::GFP::rab-10 Cbunc-119(+)];LMP-1::mCherry* | This study |
| HJZ108 | *pwIs170[vha6p::GFP::rab-7 Cbunc-119(+)];LMP-1::mCherry* | This study |
| HJZ109 | *qxIs520[pvha-6::laat-1::GFP Cbunc-119(+)];pwIs414[vha-6::RFP::rab-10, Cbunc-119(+)]* | This study |
| HJZ110 | *qxIs520[pvha-6::laat-1::GFP Cbunc-119(+)];pwIs429[pvha-6::mCherry::rab-7 Cbunc-119(+)]* | This study |
| HJZ111 | *pwIs170[vha6p::GFP::rab-7 Cbunc-119(+)];pwIs429[pvha-6::mCherry::rab-7 Cbunc-119(+)]* | This study |
| HJZ112 | *adIs2122[plgg-1::GFP::lgg-1 rol-6(su1006)];pwIs429[pvha-6::mCherry::rab-7 Cbunc-119(+)]* | This study |
| HJZ113 | *adIs2122[plgg-1::GFP::lgg-1 rol-6(su1006)];LMP-1::mCherry* | This study |
| HJZ114 | *adIs2122[plgg-1::GFP::lgg-1 rol-6(su1006)];pwIs414[vha-6::RFP::rab-10, Cbunc-119(+)]* | This study |
| HJZ115 | *atg-18(gk378);bpIs322[flag::let-363]* | This study |
| HJZ116 | *atg-18(gk378);fgEx12[pact-5::act-5::GFP rol-6(su1006)]* | This study |
| HJZ117 | *atg-18(gk378);dkIs37[pact-5::GFP::pgp-1 unc-119(+)]* | This study |
| HJZ118 | *atg-18(gk378);jcIs1[ajm-1::GFP unc-29(+) rol-6(su1006)] IV.* | This study |
| HJZ119 | *atg-18(gk378);fgEx13[perm-1::erm-1::GFP rol-6(su1006)]* | This study |
| HJZ120 | *atg-18(gk378);adIs2122[plgg-1::GFP::lgg-1 rol-6(su1006)]* | This study |
| HJZ121 | *atg-18(gk378);pwIs206[vha6p::GFP::rab-10 Cbunc-119(+)]* | This study |
| HJZ122 | *atg-18(gk378);pwIs69[vha6p::GFP::rab-11 Cbunc-119(+)]* | This study |
| HJZ123 | *atg-18(gk378);pwIs170[vha6p::GFP::rab-7 Cbunc-119(+)];pwIs429[pvha-6::mCherry::rab-7 Cbunc-119(+)]* | This study |
| HJZ124 | *atg-18(gk378);adIs2122[plgg-1::GFP::lgg-1 rol-6(su1006)];pwIs429[pvha-6::mCherry::rab-7 Cbunc-119(+)]* | This study |
| HJZ125 | *atg-18(gk378);zIs356[daf-16p::daf-16a/b::GFP rol-6(su1006)]* | This study |
| HJZ126 | *daf-2(e1370);atg-18(gk378)* | This study |
| HJZ127 | *daf-2(e1370);bpIs322[flag::let-363]* | This study |
| HJZ128 | *daf-2(e1370);adIs2122[plgg-1::GFP::lgg-1 rol-6(su1006)]* | This study |
| HJZ129 | *daf-2(e1370);fgEx12[pact-5::act-5::GFP rol-6(su1006)]* | This study |
| HJZ130 | *daf-2(e1370);dkIs37[pact-5::GFP::pgp-1 unc-119(+)]* | This study |
| HJZ131 | *daf-2(e1370);fgEx13[perm-1::erm-1::GFP rol-6(su1006)]* | This study |
| HJZ132 | *bpIs322[flag::let-363];adIs2122[plgg-1::GFP::lgg-1 rol-6(su1006)]* | This study |

^1. Göbel, V., et al., Lumen morphogenesis in^ *^C. elegans^* ^requires the membrane-cytoskeleton linker erm-1. Developmental cell, 2004. 6(6): p. 865-873.^

^2. Zhang, H., et al., Apicobasal domain identities of expanding tubular membranes depend on glycosphingolipid biosynthesis. Nature cell biology, 2011. 13(10): p. 1189-1201.^

^3. Kang, J., et al., Essential roles of snap-29 in^ *^C. elegans^*^. Developmental biology, 2011. 355(1): p. 77-88.^

^4. Solinger, J.A., et al., FERARI is required for Rab11-dependent endocytic recycling. Nature cell biology, 2020. 22(2): p. 213-224.^

^5. Guo, B. et al. Genome‐wide screen identifies signaling pathways that regulate autophagy during^ *^Caenorhabditis elegans^* ^development. EMBO reports, 2014. 15: p. 705-713.^

^6. Zheng H, Yuan C, Zhang H, Chen Y, Zhang H. The tissue-and developmental stage-specific involvement of autophagy genes in aggrephagy. Autophagy 2020; 16:589-99.^

^7. Zhang, G., et al., mTOR regulates phase separation of PGL granules to modulate their autophagic degradation. Cell, 2018. 174(6): p. 1492-1506.^

^8. Li, Z., et al., A collection of toolkit strains reveals distinct localization and dynamics of membrane-associated transcripts in epithelia. Cell Reports, 2021. 35(5): p. 109072.^

^9. Li, Y., et al., M05B5. 4 (Lysosomal phospholipase A2) promotes disintegration of autophagic vesicles to maintain^ *^C. elegans^* ^development. Autophagy, 2021.^

**Table S3.** Oligonucleotides used in this study.

| gRNAs for CRISPR gene editing | |
| --- | --- |
| For *rike-1(syb1165)* | CCTCTTTTCAGACAAGACGGGTC |
|  | AGACAAGACGGGTCAACAGTTGG |
|  | CCGGACCAATGGTTGGAGCCGGT |
|  | TCCGGACCAATGGTTGGAGCCGG |
| For *rike-1(g55t;c72a)* | GAACTCCGGAAACCTTGCATAGG |
| For *dpy-10(cn64)* | CCGCTCGTGGTGCCTATGGTAGC |
| Donor oligonucleotides for homologous repair | |
| For *rike-1(g55t;c72a)* | GAAAAAAACTTCTAAATTTCAGACACCGAACTGCGCAAACCTTGCATAGGCACACGCGGCCACTCCATACGTGAAAGTTGCAAACATCAAATGGTCTCAT |
| For *dpy-10(cn64)* | CACTTGAACTTCAATACGGCAAGATGAGAATGACTGGAAACCGTACCGCATGCGGTGCCTATGGTAGCGGAGCTTCACATGGCTTCAGACCAACAGCCTAT |
| Primers for qRT-PCR | |
| *tba-1* F | TCGTTTTCAACATGCGTGAG |
| *tba-1* R | TCCAGTGCGGATCTCATCAAC |
| *atg-2* F | CCAACTCCACTTGGCGATCT |
| *atg-2* R | CGAGTTGCTCAATTCTGCCG |
| *atg-3* F | CGATCACCTCGTCCATCACT |
| *atg-3* R | GCCGTCTTCTTCGTTGATTATTTTT |
| *atg-7* F | ACGTCATCATTCGGCATGGA |
| *atg-7* R | TTCCTGGTCGTGCAACAGTG |
| *atg-9* F | ACTCACAGTCAAAACGGGCA |
| *atg-9* R | GTAGTGAGTTTCCGGTGGCT |
| *atg-13* F | GGAACCAGAGATTGCTCCAGT |
| *atg-13* R | AGCTTCTCGGGAGGTAGAACT |
| *atg-18* F | CAGGAGCCGCAAGGAGTAAT |
| *atg-18* R | GAACCGATTGGTTGCTTGCT |
| *epg-4* F | GGAATCACTTTTGTTCGGCGT |
| *epg-4* R | GACCTGCCACAGAACCAAGA |
| *sqst-1* F | AGTCACAATCCGCAATCGGT |
| *sqst-1* R | GTCCGTTGACATCACCGTAGT |
| *bec-1* F | CAGCATCCGTTGAGGTTGGA |
| *bec-1* R | GAGCGTCAGAGCAATCATTACA |
| *vps-15* F | CAGTCGAAAATCTTGGGAGCAC |
| *vps-15* R | AATTCGCTGTGGAAACGGGT |
| *vps-34* F | GGCCACCTTCCTGATTGACTAT |
| *vps-34* R | CTTGTGCACGGGTATCAGGT |
| *rab-5* F | TGCGATTCGTCAAGGGACAA |
| *rab-5* R | GGCACACAGTTTGAGTGAGG |
| *rab-7* F | TCGGGAACCAGAAAGAAGGC |
| *rab-7* R | CCCAGATCTGAAGAGTGACGG |
| *lmp-1* F | CCAACGCTTACAAGTGCTCC |
| *lmp-1* R | GTCAGTTGGGAAAACGGTGC |
| *lgg-1* F | AACGCATCCAACTTCGTCCA |
| *lgg-1* R | TTCCTCGTGATGGTCCTGGT |
| *hsp-3* F | TCAACGGAAAGGAGCCATCC |
| *hsp-3* R | GAAGACCTGGATGGTGACGG |
| *hsp-4* F | GGAGCCATCTCGTGGAATCA |
| *hsp-4* R | GGGGTTGGGTTGGGAAAGAA |
| *hsp-6* F | AGCTATTTGGGAACCACCGT |
| *hsp-6* R | GCGATGATCTTATCTCCAGCG |
| *hsp-60* F | AAAGCAGCCAACGAGGATC |
| *hsp-60* R | CTGTGCGAACCACCTTAGT |
| *hsp-70* F | CTGTGCTGATCTTTTCCGCA |
| *hsp-70* R | AGTTGAGGTCCTTCCCATTG |
| *hsp-12.6* F | TGGAGTTGTCAATGTCCTCG |
| *hsp-12.6* R | TGGGAGGAAGTTATGGGCTTC |
